# Treatment prioritization of chemotherapy-anchored regimens after EGFR-TKI failure in EGFR-mutant NSCLC: a systematic review, pairwise meta-analysis and Bayesian network meta-analysis with probabilistic multi-criteria decision analysis

**DOI:** 10.3389/fimmu.2026.1864144

**Published:** 2026-07-31

**Authors:** Yaoyao Jing, Donghui Xing, Ao Jia, Shanshan Xu, Yixin Zhai, Xiaofang Wang, Bing Ang, Zhigang Zhao

**Affiliations:** 1 Department of Day ward, Tianjin Medical University Cancer Institute and Hospital, National Clinical Research Center for Cancer, Tianjin’s Clinical Research Center for Cancer, Key Laboratory of Cancer Prevention and Therapy, Tianjin, China; 2 Department of Medical Oncology, Tianjin First Central Hospital, School of Medicine, Nankai University, Tianjin, China; 3 Frontiers Science Center for Cell Responses, State Key Laboratory of Medicinal Chemical Biology, Key Laboratory of Bioactive Materials, Ministry of Education, and College of Life Sciences, Nankai University, Tianjin, China; 4 Department of Hematology, Tianjin Medical University Cancer Institute and Hospital, National Clinical Research Center for Cancer, Tianjin’s Clinical Research Center for Cancer, Key Laboratory of Cancer Prevention and Therapy, Tianjin, China

**Keywords:** EGFR-mutant NSCLC, EGFR-TKI resistance, meta-analysis, network meta-analysis, pMCDA

## Abstract

**Background:**

In advanced EGFR-mutant non–small cell lung cancer (NSCLC) after EGFR-TKI failure, chemotherapy alone offers limited benefit, while heterogeneous efficacy and safety across combination regimens complicate treatment selection. We systematically compared chemotherapy-anchored regimens in this setting to establish an evidence-informed treatment prioritization framework.

**Methods:**

Following PRISMA 2020 guidelines, we searched PubMed, Embase, Cochrane Library, and oncology conference for randomized controlled trials evaluating patients with advanced EGFR-mutant NSCLC progressing after EGFR-TKI therapy, comparing experimental regimens (with or without chemotherapy) against platinum-based doublet chemotherapy (chemotherapy-anchored, chemo-anchored) for efficacy and safety outcomes. Primary outcomes were progression-free survival (PFS) and overall survival (OS), analyzed using hazard ratios (HRs). Fixed-effects pairwise meta-analyses and Bayesian random-effects network meta-analyses were performed to evaluate 8 treatment strategies: chemotherapy alone (Chemo, platinum-based doublet chemotherapy); chemotherapy plus immune checkpoint inhibitors (Chemo_ICI); chemotherapy plus anti-angiogenic therapy (Chemo_Antiangio); chemotherapy plus immune checkpoint inhibitors and anti-angiogenic therapy (Chemo_ICI_Antiangio); chemotherapy plus ivonescimab (Chemo_Ivo); sacTMT (sacituzumab tirumotecan); chemotherapy plus amivantamab (Chemo_Ami); and chemotherapy plus amivantamab and lazertinib (Chemo_Ami_Laz). Probabilistic multi-criteria decision analysis (pMCDA) integrated multi-criteria outcomes under three clinically informed weighting schemes (survival-focused, balanced, and safety-focused). All analyses were conducted using R software.

**Results:**

Eleven RCTs (n=3,606) were analyzed. In pairwise analyses versus Chemo, pooled results showed significant PFS benefit for Chemo_Ivo (HR 0.50, 95% CI 0.41–0.60), Chemo_ICI_Antiangio (HR 0.55, 95% CI 0.45–0.68), and Chemo_ICI (HR 0.77, 95% CI 0.67–0.88), while Chemo_Ivo also improved OS (HR 0.76, 95% CI 0.64–0.91). Under random-effects models, HR point estimates remained identical; significance was maintained in overall analyses but lost in several subgroups as confidence intervals encompassed 1. In network meta-analysis, Chemo_Ami_Laz (HR 0.44, 95% CrI 0.32–0.61), Chemo_Ami (HR 0.48, 95% CrI 0.33–0.70), sac-TMT (HR 0.49, 95% CrI 0.35–0.68), and Chemo_Ivo (HR 0.49, 95% CrI 0.38–0.64) showed the greatest PFS benefit versus Chemo. For OS, only sac-TMT (HR 0.60, 95% CrI 0.41–0.90) and Chemo_Ivo (HR 0.76, 95% CrI 0.60–0.98) were associated with a significant survival advantage. Chemo_Ami_Laz (OR 12.06, 95% CrI 3.64–53.50) and Chemo_Ami (OR 3.71, 95% CrI 1.12–12.19) were associated with a higher risk of grade ≥3 TRAEs. In pMCDA integrating efficacy and safety, sac-TMT, Chemo_Ami, and Chemo_Ivo consistently ranked as the top three strategies across all weighting schemes.

**Conclusion:**

Integrating efficacy and safety, sac-TMT, Chemo_Ami, and Chemo_Ivo appear to be the most favorable treatment options after EGFR-TKI failure in EGFR-mutant NSCLC.

**Systematic review registration:**

https://www.crd.york.ac.uk/PROSPERO/, identifier CRD420251268510.

## 1 Introduction

Epidermal growth factor receptor (*EGFR*) mutations are major driver alterations in non-small cell lung cancer (NSCLC) (1, 2), with a high prevalence of 15%–25% in Western populations (3, 4) and up to 40%–55% in Asian countries (5, 6). For treatment-naïve individuals harboring advanced EGFR-mutant NSCLC, initial therapy utilizing first- or second-generation inhibitors yields an average time to progression of 10 to 14 months (7–9), whereas third-generation counterparts substantially extend this landmark to a median of 18.9 months (10). Although front-line TKIs achieve remarkable initial objective responses, the development of acquired resistance remains virtually inescapable for most individuals. Following subsequent therapeutic failure with a first- or second-generation EGFR-TKI, the secondary *EGFR* T790M resistance mutation is identified in approximately 50% of this population (11–15).Ultimately, regardless of the underlying molecular mechanisms, an overwhelming majority of these patients will eventually face subsequent aggressive disease progression and high mortality rates, which strongly highlights an urgent and unmet medical need for more effective further therapeutic strategies.

In recent years, a multitude of novel therapeutic designs have emerged to optimize survival post *EGFR*-TKI failure, with most landmark trials assessing these experimental regimens against conventional platinum-based doublet chemotherapy as the control arm. Specifically, approaches integrating amivantamab, an EGFR–MET bispecific antibody (16, 17), have demonstrated substantial superiority in disease control relative to traditional chemotherapy, earning a recommendation within the updated National Comprehensive Cancer Network (NCCN) guidelines (18). Furthermore, modern biological configurations like ivonescimab (a PD-1/VEGF bispecific antibody) represent an innovative mechanistically driven approach that merges anti-angiogenic properties with immunotherapy, yielding highly encouraging updates compared to standard cytotoxic backbones in the HARMONI and HARMONI-A investigations (19–21). Concurrently, antibody–drug conjugates (ADCs) have advanced as highly potent options; sacituzumab tirumotecan (sac-TMT), a TROP-2–directed ADC, has recently showcased statistically meaningful extensions in both progression-free survival (PFS) and overall survival (OS) when matched against traditional chemotherapy control cohorts (22).

Despite these regulatory milestones, randomized evidence utilizing platinum-based doublets as the comparative anchor reveals striking inconsistencies across diverse efficacy and safety outcomes. For instance, evaluating immunotherapy-chemotherapy combinations against chemotherapy alone has unveiled substantial clinical heterogeneity, spanning from the negative results of KEYNOTE-789 (23) and CheckMate-722 (24) to the positive outcomes observed in ORIENT-31 (25, 26). From a tumor biology perspective, *EGFR*-mutant malignancies inherently present an un-inflamed, “immune-cold” microenvironment with minimal T-cell infiltration, which explains why simply adding immune checkpoint blockers to chemotherapy often fails (27). Conversely, incorporating anti-angiogenic components creates a synergetic cascade; inhibiting VEGF leads to regularizing abnormal tumor vasculature, which fundamentally enhances chemotherapy-induced antigen shedding and facilitates cytotoxic lymphocyte recruitment into the tumor bed (28). This biological synergy explains the clinical success of intensive quadruplet therapies over anti-angiogenic chemotherapy in distinct cohorts of IMpower150 (29, 30), though such mechanistic translation remains volatile, as evidenced by the conflicting negative findings of IMpower151 (31) and localized data from subgroup analyses of the APPLE trial (32). Consequently, because prolonged progression-free indices frequently fail to translate into true overall survival updates and can be compromised by distinct toxic profiles, frontline clinicians are forced to navigate highly fragmented, un-synthesized evidence when selecting options against the standard chemo-anchored reference.

This combination of fragmented randomized evidence and un-synthesized clinical trade-offs highlights a knowledge gap regarding optimal patient management. To address this, we hypothesized that distinct chemo-anchored strategies exhibit divergent risk-benefit profiles shaped by their therapeutic composition, necessitating a structured multi-dimensional evaluation. Consequently, we executed a pairwise meta-analysis alongside a Bayesian network meta-analysis to thoroughly analyze the safety and efficacy parameters of various comparative regimens among patients with *EGFR*-mutant NSCLC following TKI failure, utilizing platinum doublet chemotherapy as the standardized reference backbone. A Bayesian methodology was specifically chosen because its probabilistic architecture enables the simultaneous ranking of multiple interventions and effectively integrates indirect evidence across separate clinical trials. To extend our interpretive depth beyond independent outcome metrics, we further implemented a probabilistic multi-criteria decision analysis (pMCDA) (33, 34) This joint framework was not intended to dictate inflexible treatment recommendations or erect a rigid therapeutic hierarchy, but rather to systematically visualize how varying clinical priorities dynamically influence the positioning of available regimens in the face of heterogeneous evidence.

## 2 Methods

This systematic review, pairwise meta-analysis and network meta-analysis was performed in compliance with the Preferred Reporting Items for Systematic Reviews and Meta-Analyses (PRISMA) guidelines (**Supplementary Table 1**). The study protocol was prospectively registered with the International Prospective Register of Systematic Reviews (PROSPERO; CRD420251268510).

### 2.1 Literature searching strategy

We systematically searched PubMed, Embase, and the Cochrane Library from their inception to December 19, 2025, using combinations of the terms “non–small cell lung carcinoma,” “EGFR,” “PD-1,” “PD-L1,” and “immune checkpoint inhibitor” (**Supplementary Table 2**). In addition, to maximize the completeness of evidence identification, we manually screened abstracts and presentations from major international oncology conferences, including the American Society of Clinical Oncology (ASCO) Annual Meeting, the European Society for Medical Oncology (ESMO) Congress, the American Association for Cancer Research (AACR) Annual Meeting, the World Conference on Lung Cancer (WCLC), the European Lung Cancer Congress (ELCC), and the Society for Immunotherapy of Cancer (SITC) Annual Meeting. Study eligibility criteria were structured according to the PICOS framework:

Population (P): Advanced EGFR-mutant NSCLC progressing after prior EGFR-TKI therapy.

Intervention (I): Chemotherapy-anchored experimental regimen (with or without chemotherapy).

Comparator (C): Platinum-based doublet chemotherapy backbone.

Outcomes (O): Primary outcomes were PFS and OS; secondary outcomes included ORR, DCR, and grade ≥3 TRAEs.

Study design (S): Randomized controlled trials (RCTs) only.

### 2.2 Eligibility criteria

Eligible studies met the following criteria: (1) enrolled patients with pathologically confirmed advanced (stage III/IV or recurrent) EGFR-mutant NSCLC or reported outcomes for a prespecified EGFR-mutant subgroup within a broader trial population, with disease progression after at least one prior EGFR-TKI; (2) compared an experimental regimen (with or without chemotherapy) against a platinum-based doublet chemotherapy backbone (chemotherapy alone or combined with other agents); (3) reported at least one clinical outcome, including PFS, OS, Objective Response Rate (ORR), Disease Control Rate (DCR), or grade ≥3 treatment-related adverse events(grade ≥3 TRAEs); and (4) adopted a randomized controlled design; and (5) were published as peer-reviewed full-text articles or high-quality international oncology conference abstracts. Studies were excluded if they were conducted in the adjuvant or neoadjuvant setting, focused on first-line treatment, were retrospective or real-world studies, or were phase I clinical trials, or lacked accessible data from main publications or conference updates.

### 2.3 Data extraction and quality assessment

Two investigators (YJ and DX) independently performed study screening and data extraction using a standardized data collection form, in accordance with the guidelines of the Cochrane Collaboration. Discrepancies were resolved through discussion or, when necessary, by consultation with a third author (ZZ). Extracted data included study characteristics (trial identifier, study phase, publication year, region, and sample size), treatment regimens, and outcomes, including hazard ratios (HRs) with corresponding 95% confidence intervals (CIs) for PFS and OS, as well as ORR, DCR, and the incidence of grade ≥3 TRAEs. Study quality and risk of bias assessments were performed for all included trials. Risk of bias assessment was explicitly performed by the same two investigators using the Cochrane Handbook for Systematic Reviews of Interventions (RoB1) (35). Each included trial was comprehensively evaluated across specific domains and categorized into distinct risk of bias categories: low risk of bias, unclear risk, or high risk of bias, in strict accordance with the Cochrane guidelines.

### 2.4 Statistical analysis

All statistical analyses were performed using R software (version 4.4.3). Pairwise meta-analysis was performed using the meta package (Version 8.1.0) in R for the primary outcomes (PFS and OS), and selectively applied to direct head-to-head comparisons in which chemotherapy alone served as the control arm. Effect estimates from a single randomized controlled trial were presented without pooling. Conversely, when two or more trials were available, pooled estimates were calculated using a fixed-effects model. Concurrently, random-effects model were also performed for sensitivity analyses. Statistical heterogeneity was assessed using the Cochran Q test and the I² statistic (36).

Bayesian network meta-analysis (NMA) was performed using the gemtc package (Version 1.1.0) in R, which interacts with Just Another Gibbs Sampler (JAGS). For time-to-event outcomes (PFS and OS), Bayesian random-effects NMA was performed based on the logHR and its standard error (SE), assuming a normal likelihood with an identity link function. For binary outcomes (ORR, DCR, and grade ≥3 TRAEs), odds ratios (ORs) with 95%CI were analyzed using a binomial likelihood with a logit link. Non-informative prior distributions were specified for all model parameters. Specifically, a vague normal prior (~N (0, 10,000)) was assigned to treatment effect parameters (log hazard ratios and log odds ratios), and a uniform prior (~Uniform (0, 5)) was applied to the between-study heterogeneity variance (τ 2) in random-effects models. Bayesian inference was performed using Markov chain Monte Carlo (MCMC) methods through JAGS in R, with four chains, 50,000 iterations, 20,000 burn-in iterations, and thinning of 10. Random-effects consistency models were selected as the primary analytical framework. Model fit was evaluated using the Deviance Information Criterion (DIC),with a fixed-effects model serving as a sensitivity analysis (DIC difference <5 favored the random-effects model). Convergence was evaluated by visual inspection of posterior trace plots and density plots, as well as by the Gelman–Rubin diagnostic, with potential scale reduction factors (PSRF) close to 1 indicating adequate convergence. Global and local inconsistencies were examined using the unrelated mean effects (UME) model and the node-splitting approach, respectively (37, 38). Comparison-adjusted funnel plots were constructed to explore potential small-study effects and publication bias. Relative treatment rankings were quantified via surface under the cumulative ranking curve (SUCRA) values (39).

A pMCDA was further conducted to integrate multiple efficacy and safety outcomes and to support evidence-informed treatment prioritization (40). Posterior estimates from the NMA were transformed into normal distributions based on the mean and standard deviation of the log HRs (for PFS and OS) or log ORs (for ORR, DCR, and grade ≥3 adverse events), and Monte Carlo simulations with 20,000 iterations were performed. All outcomes were rescaled to a 0–1 utility scale, where higher values indicated better performance. Composite scores were calculated as weighted sums of the utilities across outcomes, according to the following formula:


$$U_{j}=\sum_{i=1}^{k}w_{i}\times u_{ij}$$

where $U_{j}$ denotes the composite score of treatment j, $w_{i}$ is the predefined weight of outcome i, and $u_{ij}$ is the normalized utility value of treatment j for outcome i. Three predefined weighting schemes were applied to reflect different clinical priorities (survival-focused, balanced, and safety-focused). The weighting schemes were predefined *a priori* and determined by consensus between two experienced clinical oncologists (ZZ and BA). For each scheme, the mean composite score, its 95% credible interval (CrI), and the probability of each treatment being the optimal option were estimated.

### 2.5 Evaluation of the quality of evidence

The certainty of evidence for each outcome was appraised using the Grading of Recommendations Assessment, Development and Evaluation (GRADE) framework adapted for network meta-analysis. In accordance with the standard GRADE development guidelines, evidence derived from RCTs started at a “high” certainty level and was systematically evaluated for downgrading based on six structural domains: within-study bias (risk of bias), reporting bias, indirectness, imprecision, heterogeneity, and incoherence. The final certainty of evidence for each treatment comparison was categorized into four tiers: high, moderate, low, or very low.

## 3 Results

### 3.1 PRISMA study search results

**Figure 1:**
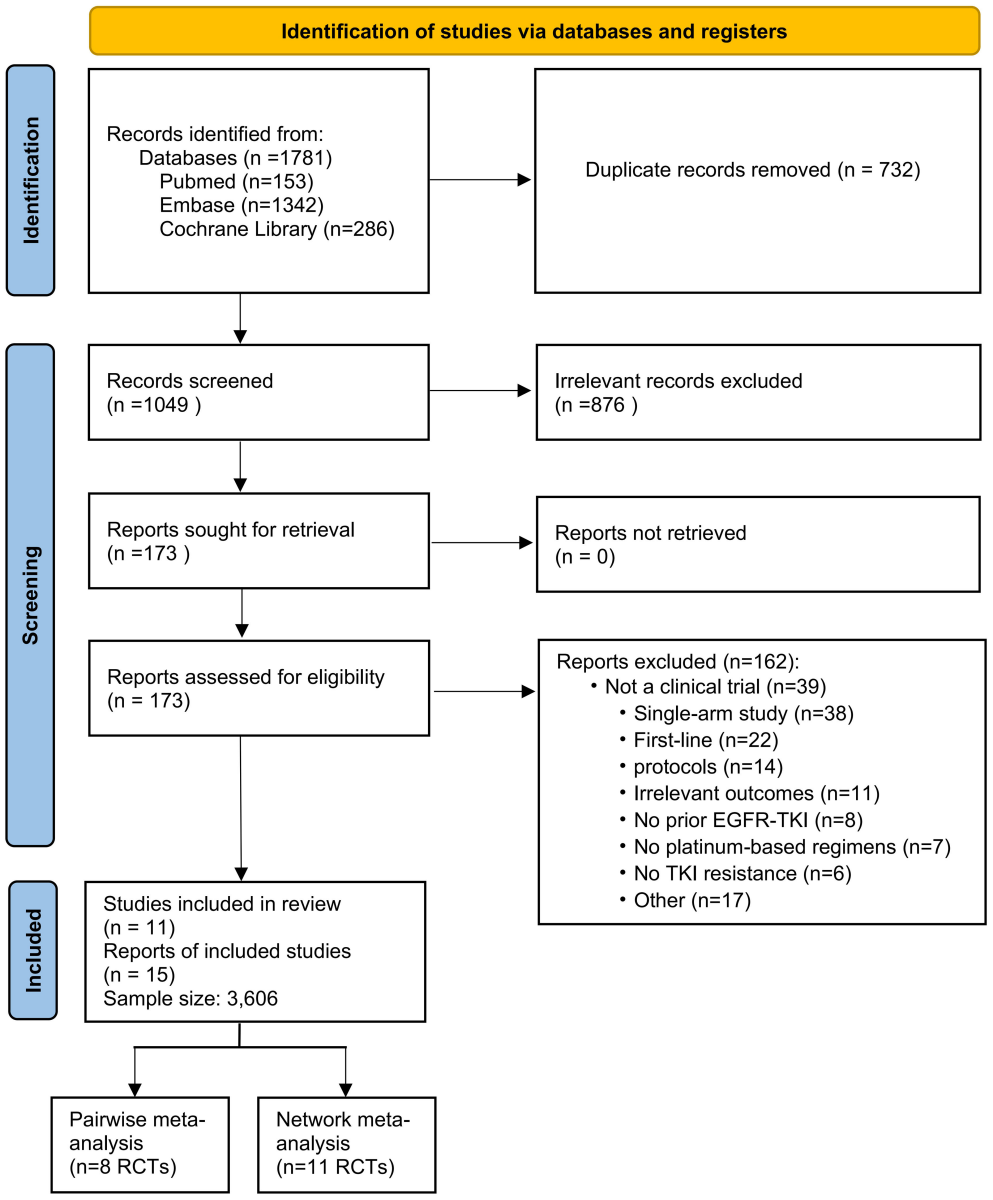
PRISMA 2020 flow diagram. TKI, tyrosine-kinase inhibitor.

The systematic database search initially identified a total of 1,781 records, including 153 from PubMed, 1,342 from Embase, and 286 from the Cochrane Library. After the systematic removal of 732 duplicated records, 1,049 studies underwent initial screening based on titles and abstracts, after which 173 potentially relevant full-text articles were retrieved and meticulously assessed for eligibility. During the final full-text evaluation stage, a total of 162 articles were explicitly excluded with well-defined reasons, including 39 records that were not a clinical trial, 38 single-arm studies, 22 evaluating regimens as a first-line treatment, 14 study protocols, 11 reporting irrelevant outcomes, 8 enrolling populations with no prior EGFR-TKI exposure, 7 utilizing no platinum-based regimens, 6 including patients without established TKI resistance, and 17 for other miscellaneous reasons. Ultimately, 11 phase III randomized controlled trials (RCTs) (16, 17, 19–21, 23–26, 29–32, 41, 42). with an aggregate sample size of 3,606 patients fulfilled all criteria and were included in the final synthesis, where all 11 RCTs contributed data to the network meta-analysis, while 8 RCTs (16, 17, 19–21, 23–26, 41, 42) were selectively utilized for the frequentist pairwise meta-analysis (**Figure 1**).

### 3.2 Study and population characteristics

The 11 included phase III RCTs (16, 17, 19–21, 23–26, 29–32, 41, 42) randomized a total of 3,606 patients with pathologically confirmed advanced *EGFR*-mutant NSCLC who experienced disease progression following prior EGFR-TKI therapy. To ensure an up-to-date and robust evidence base, recently reported clinical updates were fully incorporated, including newly available OS data from the IMpower151 trial (31), updated outcomes from the HARMONI and HARMONI-A trials evaluating the novel anti-PD-1/VEGF bispecific antibody ivonescimab combined with chemotherapy (19–21), and latest data from trials investigating sac-TMT (a novel TROP-2–targeting antibody–drug conjugate) (22).

The included trials were summarized into eight chemo-anchored treatment strategies: chemotherapy alone (Chemo, platinum-based doublet chemotherapy); chemotherapy plus immune checkpoint inhibitors (Chemo_ICI); chemotherapy plus anti-angiogenic therapy (Chemo_Antiangio); chemotherapy plus immune checkpoint inhibitors and anti-angiogenic therapy (Chemo_ICI_Antiangio); chemotherapy plus ivonescimab (Chemo_Ivo); sacTMT (sacituzumab tirumotecan); chemotherapy plus amivantamab (Chemo_Ami); and chemotherapy plus amivantamab and lazertinib (Chemo_Ami_Laz). Detailed study characteristics are provided in **Supplementary Table 3**.

### 3.3 Risk of bias assessment

Risk of bias was assessed using the Cochrane risk of bias tool. Six trials were judged to be at high risk of bias, primarily due to open-label designs with inadequate blinding, which constituted the main source of bias. The HARMONI trial was additionally rated as high risk because outcome data were derived from conference abstracts (**Supplementary Figure 1**).

### 3.4 Network topology and quality assessment

For the Bayesian network meta-analysis, model convergence was achieved for PFS, as indicated by well-mixed trace plots, stable posterior density distributions, and Gelman–Rubin potential scale reduction factors (PSRFs) close to 1 (**Supplementary Figure 3**). Similar convergence patterns were observed for the other outcomes. Between-study heterogeneity was low across outcomes (**Supplementary Table 4**), indicating no substantial variability. Local consistency was supported by node-splitting analyses (**Supplementary Table 5**) and global consistency was supported by similar fit of consistency and inconsistency models (**Supplementary Table 6**). Sensitivity analyses showed robust results, with similar estimates obtained from consistency random-effects and fixed-effects models (**Supplementary Table 6**). Comparison-adjusted funnel plots showed no obvious evidence of publication bias (**Supplementary Figure 4**).

### 3.5 Efficacy and safety outcomes

#### 3.5.1 PFS

For PFS, pairwise meta-analyses were conducted for selected treatment contrasts with chemotherapy alone as the common comparator for PFS in 8 RCTs (16, 17, 19–21, 23–26, 41, 42). Results of direct comparisons are presented in **Supplementary Figure 2**. In the fixed-effect framework, pooled analyses showed that Chemo_Ivo (HR 0.50, 95% CI 0.41–0.60), Chemo_ICI_Antiangio (HR 0.55, 95% CI 0.45–0.68), and Chemo_ICI (HR 0.77, 95% CI 0.67–0.88) significantly improved PFS compared with chemotherapy. Comparisons informed by individual randomized trials, including Chemo_Ami (HR 0.48, 95% CI 0.36–0.64), Chemo_Ami_Laz (HR 0.44, 95% CI 0.35–0.56), and sac-TMT (HR 0.49, 95% CI 0.39–0.62), showed similar benefits. When both statistical models were projected simultaneously, the hazard ratio point estimates between the fixed-effect and random-effects frameworks remained highly comparable across all comparisons. However, the random-effects model yielded wider 95% confidence intervals, under which Chemo_ICI maintained its statistical significance, whereas Chemo_ICI_antiangio and Chemo_Ivo did not maintain statistical significance as their confidence interval expanded to encompass 1 (HR 0.55, 95% CI: 0.16–1.87) and (HR 0.50, 95% CI: 0.23–1.06), respectively.

**Figure 2:**
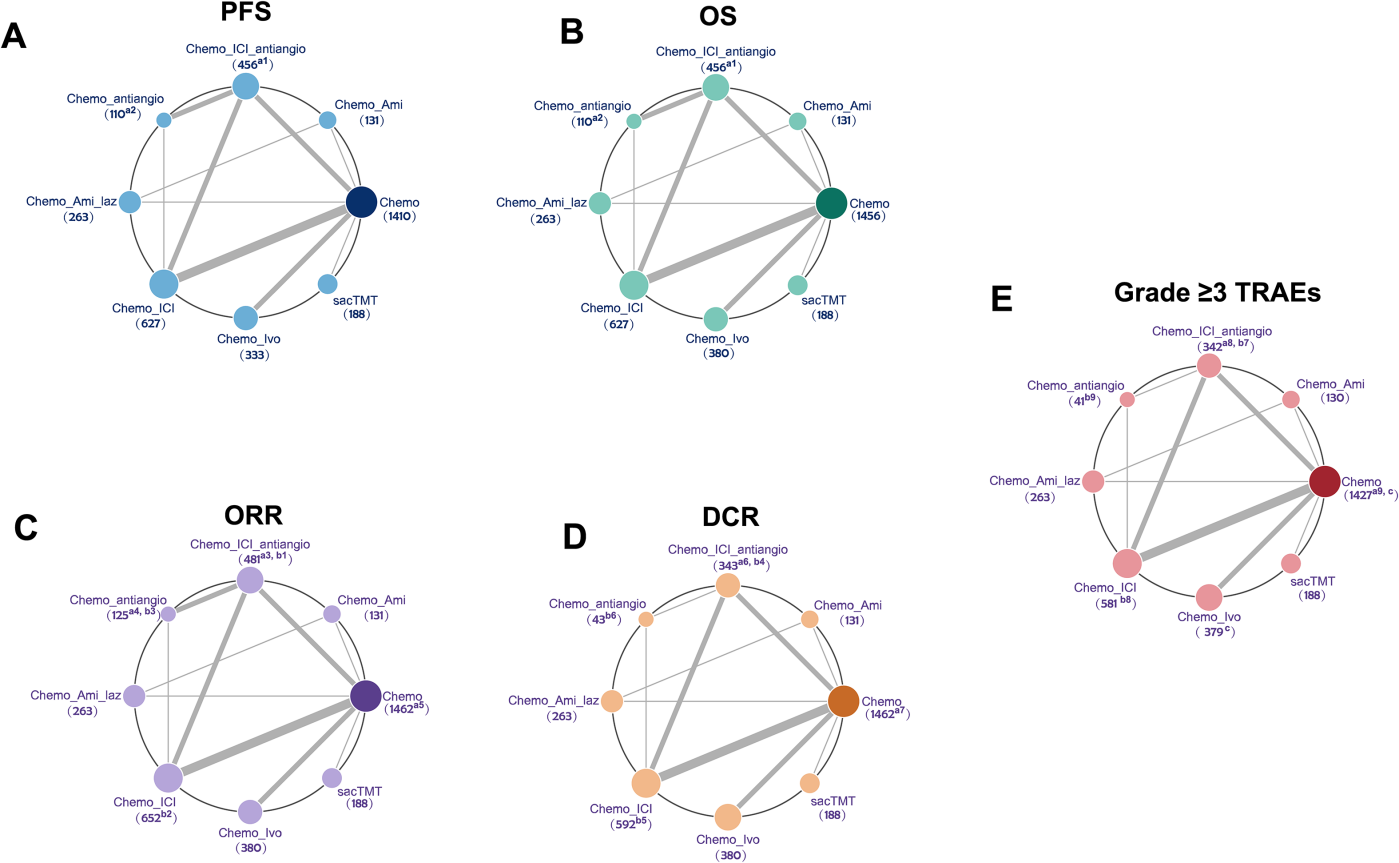
Network plot illustrating treatment comparisons among the included trials. **(A)** Progression-free survival. **(B)** Overall survival. **(C)** Objective response rate. **(D)** Disease control rate. **(E)** Grade≥3 treatment-grade adverse events. ^a^Including patients with ALK rearrangements (numbers of patients in a1–a9 were 2, 3, 9, 3, 6, 7, 6, 7, and 6, respectively). ^b^Including EGFR-positive patients who had not received any prior tyrosine kinase inhibitor (numbers of patients in b1–b9 were 18, 25, 17, 12, 17, 17, 11, 11, and 13, respectively). ^c^Including 161 patients with treatment-emergent adverse events (TEAEs). Lines represent treatment comparisons informed by randomized trials, with line thickness proportional to the number of contributing studies. Comparisons may arise from either direct head-to-head trials or multi-arm trials with shared comparators. Indirect comparisons are estimated by synthesizing all available evidence within the network. Chemo, platinum-based doublet chemotherapy. ICI, immune checkpoint inhibitor. Chemo_ICI, chemotherapy plus ICI; Chemo_Antiangio, chemotherapy plus antiangiogenesis; Chemo_ICI_antiangio, chemotherapy plus antiangiogenesis plus ICI; Chemo_Ivo,chemotherapy plus Ivonescimab; sacTMT, Sacituzumab Tirumotecan; Chemo_Ami, chemotherapy plus Amivantamab; Chemo_Ami_Laz, chemotherapy plus Amivantamab plus lazertinib.

**Figure 3:**
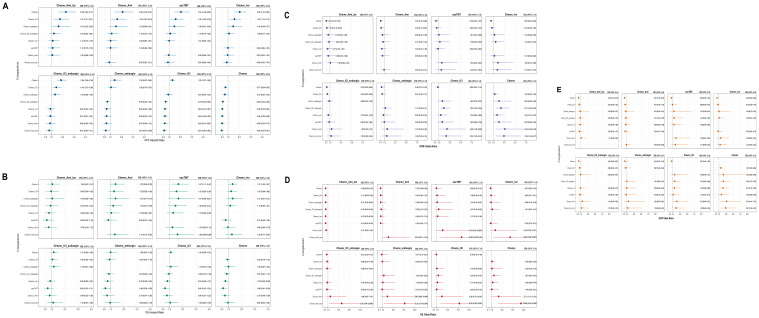
Forest plot of hazard ratios or odds ratios across different treatment regimens. **(A)** Progression-free survival. **(B)** Overall survival. **(C)** Objective response rate. **(D)** Grade≥3 treatment-grade adverse events. **(E)** Disease Controlled Rate. *For visualization purposes, the upper limit of the 95% credible interval was capped at 13.00; the complete values are provided in the league table in the **Supplementary Material**. ^※^OS data were not mature for the Chemo_Ami_Laz versus Chemo arm in MARIPOSA-2, for the Western population in HARMONI, and in IMpower151. Chemo, platinum-based doublet chemotherapy. ICI, immune checkpoint inhibitor; Chemo_ICI, chemotherapy plus ICI; Chemo_Antiangio, chemotherapy plus antiangiogenesis; Chemo_ICI_antiangio, chemotherapy plus antiangiogenesis plus ICI; Chemo_Ivo, chemotherapy plus Ivonescimab; sacTMT, Sacituzumab Tirumotecan; Chemo_Ami, chemotherapy plus Amivantamab; Chemo_Ami_Laz, chemotherapy plus Amivantamab plus lazertinib; HR, Hazard ratio; OR, Odds Ratio; CrI, Credible Interval.

In the network meta-analysis, all 11 RCTs (16, 17, 19–21, 23–26, 29–32, 41, 42) contributed to the analyses of PFS (**Figure 2**). Chemo_Ami_Laz (HR 0.44 [95% CrI 0.32–0.61]), Chemo_Ami (HR 0.48 [0.33–0.70]), sac-TMT (HR 0.49 [0.35–0.68]), and Chemo_Ivo (HR 0.49 [0.38–0.64]) showed favorable efficacy and were all significantly superior to Chemo, each associated with a reduction of more than 50% in the risk of disease progression. In addition, Chemo_ICI_antiangio (HR 0.54 [0.43–0.68]) and Chemo_ICI (HR 0.77 [0.64–0.93]) were also associated with reduced progression risk compared with Chemo. However, no statistically differences were observed among the four top-performing strategies for PFS (Chemo_Ami_Laz, Chemo_Ami, sac-TMT, and Chemo_Ivo), although all four were significantly superior to Chemo_ICI (**Figure 3A**).

Based on SUCRA-derived ranking, Chemo_Ami_Laz ranked first for PFS (SUCRA = 0.845) (**Supplementary Table 5**; **Supplementary Figures 6**, **7**).

#### 3.5.2 OS

For OS, pairwise meta-analyses were also conducted for selected treatment contrasts with chemotherapy alone as the common comparator for OS in 8 RCTs (16, 17, 19–21, 23–26, 41, 42). Results of direct comparisons are presented in **Supplementary Figure 2**. In the fixed-effect framework, pooled results indicated a significant survival benefit for Chemo_Ivo (HR 0.76, 95% CI 0.64–0.91). Consistent improvements were also observed in pairwise comparisons informed by individual randomized trials for Chemo_Ami (HR 0.73, 95% CI 0.54–0.99) and sac-TMT (HR 0.60, 95% CI 0.44–0.82). When both statistical models were projected simultaneously, the hazard ratio point estimates between the fixed-effect and random-effects frameworks remained identical across all pooled comparisons. However, the random-effects model yielded wider 95% confidence intervals, under which none of the pooled treatment regimens maintained statistical significance as their confidence intervals expanded to encompass 1: Chemo_ICI (HR 0.86, 95% CI: 0.71–1.04), Chemo_ICI_antiangio (HR 0.98, 95% CI: 0.92–1.05), and Chemo_Ivo (HR 0.76, 95% CI: 0.50–1.16), respectively.

In the network meta-analysis, all 11 RCTs (16, 17, 19–21, 23–26, 29–32, 41, 42) contributed to the analyses of OS (**Figure 2**). Compared with Chemo, sac-TMT (HR 0.60 [95% CrI 0.41–0.90]) and Chemo_Ivo (HR 0.76 [0.60–0.98]) were the only two strategies associated with a statistically significant survival benefit. Although sac-TMT showed a numerically lower risk of death than Chemo_Ivo, the difference was not statistically significant (HR 0.79 [0.49–1.26]) (**Figure 3B**).

Based on SUCRA-derived ranking, sac-TMT ranked first for OS (SUCRA = 0.918) (**Supplementary Table 5**; **Supplementary Figures 6**, **7**).

#### 3.5.3 Tumor response outcomes

For tumor response outcomes, all 11 RCTs contributed to the analyses of ORR, whereas 9 RCTs (16, 17, 19–21, 23–26, 29, 30, 41, 42) were included in the analyses of DCR, with IMpower151 and APPLE not contributing data for these outcomes (**Figure 2**). In the network meta-analysis, Chemo_ICI_antiangio, Chemo_Ivo, Chemo_Ami, and Chemo_Ami_Laz were associated with significantly improved ORR and DCR compared with Chemo. Specifically, Chemo_ICI_antiangio (ORR: OR 2.41 [95% CrI 1.53–3.95]; DCR: OR 2.26 [1.27–4.25]), Chemo_Ivo (ORR: OR 1.69 [1.01–2.87]; DCR: OR 2.15 [1.22–3.85]), Chemo_Ami (ORR: OR 3.13 [1.47–6.72]; DCR: OR 3.18 [1.44–7.15]), and Chemo_Ami_Laz (ORR: OR 2.95 [1.42–6.07]; DCR: OR 2.87 [1.39–5.91]) all showed favorable tumor response outcomes. No statistically significant differences were observed among these four strategies for either ORR or DCR (**Figures 3C, E**).

Based on SUCRA-derived ranking, Chemo_Ami ranked first for both ORR (SUCRA = 0.835) and DCR (SUCRA = 0.817) (**Supplementary Table 5** **Supplementary Figures 6**, **7**).

#### 3.5.4 Safety outcomes

For safety outcomes, 9 RCTs (16, 17, 19–21, 23–26, 29, 30, 41, 42) were included in the analyses of grade ≥3 TRAEs, with IMpower151 and APPLE not contributing data for this outcome (**Figure 2**). Notably, while most trials reported TRAEs, the HARMONi-A trial utilized treatment-emergent adverse events (TEAEs) as a proxy. In the network meta-analysis, the risk of grade ≥3 TRAEs differed across treatment strategies. Compared with Chemo, Chemo_Ami_Laz (OR 12.06 [95% CrI 3.64–53.50]) and Chemo_Ami (OR 3.71 [95% CrI 1.12–12.19]) were associated with a significantly increased risk of severe TRAEs. Chemo_Ami showed a numerically lower risk of severe TRAEs than Chemo_Ami_Laz (OR 0.31 [95% CrI 0.09–1.04]), although the difference did not reach statistical significance (**Figure 3D**; **Supplementary Figure 5**). The complete results of all pairwise comparisons derived from the network meta-analysis are summarized in the league tables provided in the Supplementary Material (**Supplementary Figure 5**).

Based on SUCRA-derived ranking, Chemo_Ami_Laz showed the lowest safety ranking (SUCRA = 0.010) (**Supplementary Table 5**; **Supplementary Figures 6**, **7**).

In a sensitivity analysis excluding the HARMONi-A trial, the SUCRA-derived safety hierarchies remained unchanged. However, regarding individual comparisons versus chemotherapy, Chemo_Ami lost its statistical significance (OR 3.70, 95% CrI: 0.80–16.69), while Chemo_Ami_laz maintained a significantly increased risk of severe adverse events (OR 11.96, 95% CrI: 2.63–53.36) (**Supplementary Figure 8**).

Furthermore, independent network meta-analyses were performed for six specific safety sub-endpoints (**Supplementary Figure 9**). Depending on standard literature reporting constraints, the number of informative trials integrated into each sub-network ranged from 6 to 8 out of the 9 trials utilized in the overall safety dataset. Specifically, both Chemo and Chemo_ICI showed a significantly lower risk of treatment-related deaths compared with Chemo_ICI_antiangio [(OR 0.10, 95% CrI: 0.01, 0.69) and (OR 0.11, 95% CrI: 0.01, 0.92), respectively], as well as a lower risk of treatment discontinuation [(OR 0.24, 95% CrI: 0.09, 0.50) and (OR 0.40, 95% CrI: 0.18, 0.80), respectively]. Additionally, Chemo_Ami_laz was associated with a profoundly higher risk of decreased platelet count compared with sacTMT (OR 7.65, 95% CrI: 1.13, 53.95).

### 3.6 Integrated treatment ranking via pMCDA

**Figure 4:**
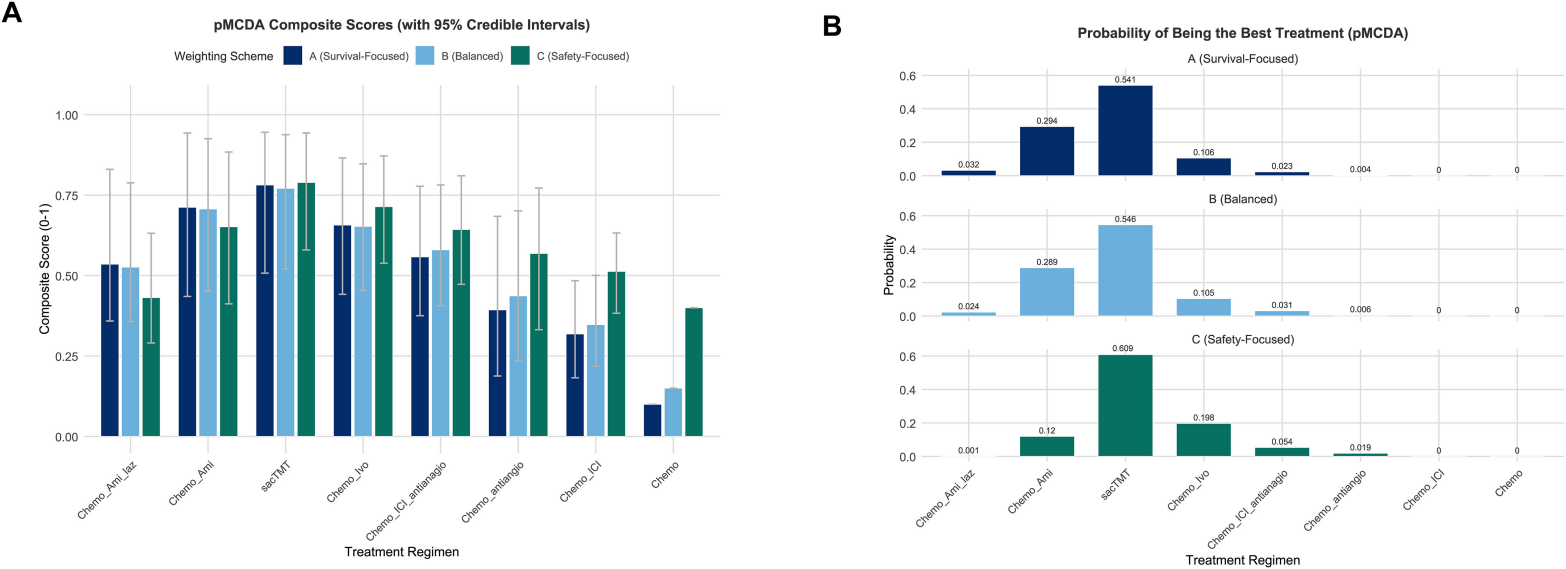
Treatment ranking based on probabilistic multi-criteria decision analysis with different weighting schemes. **(A)** Composite scores. **(B)** Probability of being the best treatment. Chemo, platinum-based doublet chemotherapy. ICI, immune checkpoint inhibitor; Chemo_ICI, chemotherapy plus ICI; Chemo_Antiangio, chemotherapy plus antiangiogenesis; Chemo_ICI_antiangio, chemotherapy plus antiangiogenesis plus ICI; Chemo_Ivo, chemotherapy plus Ivonescimab; sacTMT, Sacituzumab Tirumotecan; Chemo_Ami, chemotherapy plus Amivantamab; Chemo_Ami_Laz, chemotherapy plus Amivantamab plus lazertinib; pMCDA, probabilistic Multi-Criteria Decision Analysis.

Building upon the consistency observed between single-outcome Bayesian rankings and corresponding effect estimates, pMCDA was applied to integrate multiple clinically relevant outcomes into a composite treatment ranking. Different clinically informed weighting schemes were applied across outcomes (**Supplementary Table 8**), using three predefined alternative weighting schemes: A (survival-focused), B (balanced), and C (safety-focused). The resulting pMCDA-based composite rankings are shown in **Figure 4**. Overall, across the three weighting schemes, sac-TMT, Chemo_Ami, and Chemo_Ivo consistently ranked as the top three strategies, achieving the highest composite scores among all regimens, with mean scores of 0.782, 0.771, and 0.789 for sac-TMT; 0.712, 0.707, and 0.652 for Chemo_Ami; and 0.657, 0.653, and 0.714 for Chemo_Ivo under schemes A, B, and C, respectively (**Figure 4A**; **Supplementary Table 9**). Specifically, sac-TMT had the highest probability of being the best treatment under all three schemes (probabilities: 0.541, 0.546, and 0.609). Chemo_Ami ranked second in the probability of being the best treatment under schemes A and B (probabilities: 0.294 and 0.289), whereas Chemo_Ivo ranked second under schemes C (probability: 0.198) (**Figure 4B**).

### 3.7 GRADE assessment results

The certainty of evidence for all outcomes is summarized in **Supplementary Figure 10**. For efficacy endpoints (PFS, OS, ORR, and DCR), the majority of evaluated treatment comparisons demonstrated high-to-moderate certainty, with regimens involving sac-TMT, amivantamab combinations, and ivonescimab plus chemotherapy consistently achieving high certainty. Conversely, the evidence for Chemo-ICI-antiangio vs Chemo was graded as low certainty across all efficacy outcomes. For safety profiles (grade≥3 TRAEs), the majority of evaluated regimens maintained high certainty.

## 4 Discussion

### 4.1 Summary of main findings

This analysis addresses a central clinical dilemma after EGFR-TKI resistance: how to navigate treatment selection when multiple active regimens demonstrate meaningful but non-uniform benefits across efficacy and safety outcomes. In this Bayesian network meta-analysis of randomized trials evaluating systemic treatment strategies in this setting, multiple regimens were found to confer clinically meaningful benefits compared with chemotherapy; however, their relative performance varied substantially across endpoints. For progression-free survival, Chemo_Ami_Laz, Chemo_Ami, sac-TMT, and Chemo_Ivo consistently formed the leading efficacy tier. Importantly, these pronounced PFS advantages did not uniformly translate into overall survival benefit, as statistically significant OS improvement was confined to sac-TMT and Chemo_Ivo, highlighting heterogeneity in long-term benefit beyond early disease control.

### 4.2 Alignment with introduction hypotheses and knowledge gaps

These results directly address the critical knowledge gaps regarding post-TKI therapeutic prioritization. While forest plots and standard single-outcome Bayesian ranking metrics establish relative treatment effects for isolated endpoints, they fail to capture the multidimensional trade-offs inherent to real-world clinical decision-making, particularly when survival durability and safety must be jointly considered. For instance, while amivantamab-containing combination regimens achieved the most substantial improvements in PFS, they concurrently carried a higher incidence of grade≥3TRAEs. This is consistent with its biological rationale: as an EGFR-MET bispecific antibody, amivantamab potently shuts down hyperactivated bypass pathways (such as MET amplification) driving TKI resistance, but its concurrent systemic blockade of wild-type receptors inevitably exacerbates severe on-target dermatological and gastrointestinal toxicities (MARIPOSA-2).Conversely, the OS benefit for Chemo_Ami observed in pairwise analyses was derived from a single randomized trial and did not persist within the network meta-analysis. Collectively, these discordant endpoint-specific signals confirm our initial hypothesis that single-outcome rankings are insufficient to provide coherent therapeutic prioritization, necessitating a structured translation of heterogeneous comparative evidence into an integrated, decision-oriented framework.

### 4.3 Comparison and comprehensive analysis with previous studies

The clinical evidence evaluating subsequent systemic strategies has been highly heterogeneous across historical phase III trials. Regarding ICI additions, both KEYNOTE-789 (23) and CheckMate-722 (24) failed to demonstrate a survival benefit for ICI plus chemotherapy over chemotherapy alone, whereas ORIENT-31 (25, 26) reported significant PFS prolongation. Conflicting findings similarly emerge from trials combining chemotherapy, immunotherapy, and anti-angiogenic agents. IMpower150 (29, 30) demonstrated a clear PFS benefit for Chemo_ICI_antiangio over Chemo_antiangio, whereas IMpower151 (31) reported no statistically significant advantage for a comparable strategy. Our network meta-analysis mirrors these discrepancies, showing that adding conventional ICIs to platinum-based chemotherapy yielded a statistically significant but modest PFS improvement over chemotherapy alone, while no significant benefit was observed for Chemo_ICI_antiangio over Chemo_antiangio. This further underscores the volatility of treatment effects across ICI-based combinations.

The discordant outcomes between IMpower150 and IMpower151 also raise the possibility that the choice of chemotherapy backbone may modulate the efficacy of immunotherapy-based combinations, as IMpower150 predominantly employed carboplatin plus paclitaxel (29, 30), whereas IMpower151 largely relied on carboplatin plus pemetrexed, with approximately 97% of patients receiving pemetrexed (31). Given the distinct biological and immunomodulatory properties of taxane- and pemetrexed-based chemotherapy, differential interactions with immune checkpoint blockade cannot be excluded. Furthermore, more comprehensive immunomodulatory strategies may overcome intrinsic resistance in EGFR-mutant tumors. Ivonescimab, a bispecific antibody targeting both PD-1 and VEGF, integrates immune checkpoint blockade with anti-angiogenic modulation within a single molecule (43). Consequently, Chemo_Ivo emerged as one of the most effective regimens in our analysis, showing concurrent improvements in PFS and OS while maintaining a comparatively favorable safety profile. This pronounced efficacy suggests that dual PD-1/VEGF blockade may cooperatively modulate immune suppression and tumor angiogenesis. Biologically, the simultaneous neutralization of VEGF not only normalizes tumor vasculature to facilitate T-cell trafficking but also reverses the characteristically “cold”, immunosuppressive tumor microenvironment typically induced by EGFR-TKI resistance (28).

Concurrently, sac-TMT, a TROP2-directed antibody–drug conjugate (ADC) (42), demonstrated robust and consistent benefits in both PFS and OS compared with chemotherapy, while maintaining a favorable safety profile (ranking second for safety via SUCRA). These findings reinforce the emerging role of ADC-based strategies in the post–EGFR-TKI setting. Datopotamab deruxtecan (Dato-DXd), another TROP2-targeting ADC, has recently been incorporated into the latest NCCN guidelines based on a subgroup analysis of the phase III TROPION-Lung01 trial (18, 44). However, because the pivotal trial of Dato-DXd employed docetaxel monotherapy rather than a platinum-based doublet as the comparator, it was methodologically incompatible with our network backbone and excluded from quantitative synthesis. Nonetheless, differences in payload, linker design, and trial populations across ADC programs may contribute to distinct efficacy and tolerability profiles, positioning sac-TMT as a compelling ADC option among regimens evaluated on a consistent platinum doublet backbone. The biological superiority of this strategy relies on the widespread surface overexpression of TROP2 after TKI failure, allowing the ADC to bypass downstream intracellular resistance mutations entirely and eradicate neighboring tumor cells via a potent bystander effect (45).

### 4.4 Key findings derived from this analysis

To our knowledge, this study represents the first application of pMCDA within a network meta-analytic framework to inform treatment prioritization in patients with EGFR-TKI–resistant NSCLC. Multi-criteria decision analysis (MCDA) integrates multiple clinical criteria into a structured decision framework (33, 34, 40). While forest plots and ranking metrics derived from network meta-analysis establish relative treatment effects for individual endpoints, they do not fully capture the multidimensional trade-offs inherent to real-world clinical decision-making, particularly when efficacy, durability of benefit, and toxicity must be considered simultaneously. Probabilistic MCDA extends this framework by incorporating uncertainty in each criterion through probabilistic modeling. By jointly integrating progression-free survival, overall survival, tumor response, and grade ≥3 treatment-related adverse events under clinically plausible weighting scenarios, pMCDA integrated efficacy and safety estimates derived from the network meta-analysis across all evaluated regimens, enabling prioritization under clinically plausible weighting scenarios. Importantly, pMCDA was applied as a decision-support tool rather than a substitute for randomized evidence, preserving the primacy of effect estimates derived from forest plots. Across multiple weighting schemes reflecting different clinical priorities, sac-TMT, Chemo_Ami, and Chemo_Ivo consistently achieved the highest overall benefit–risk profiles, underscoring the robustness of these findings.

### 4.5 Evidence-informed clinical recommendations

**Figure 5:**
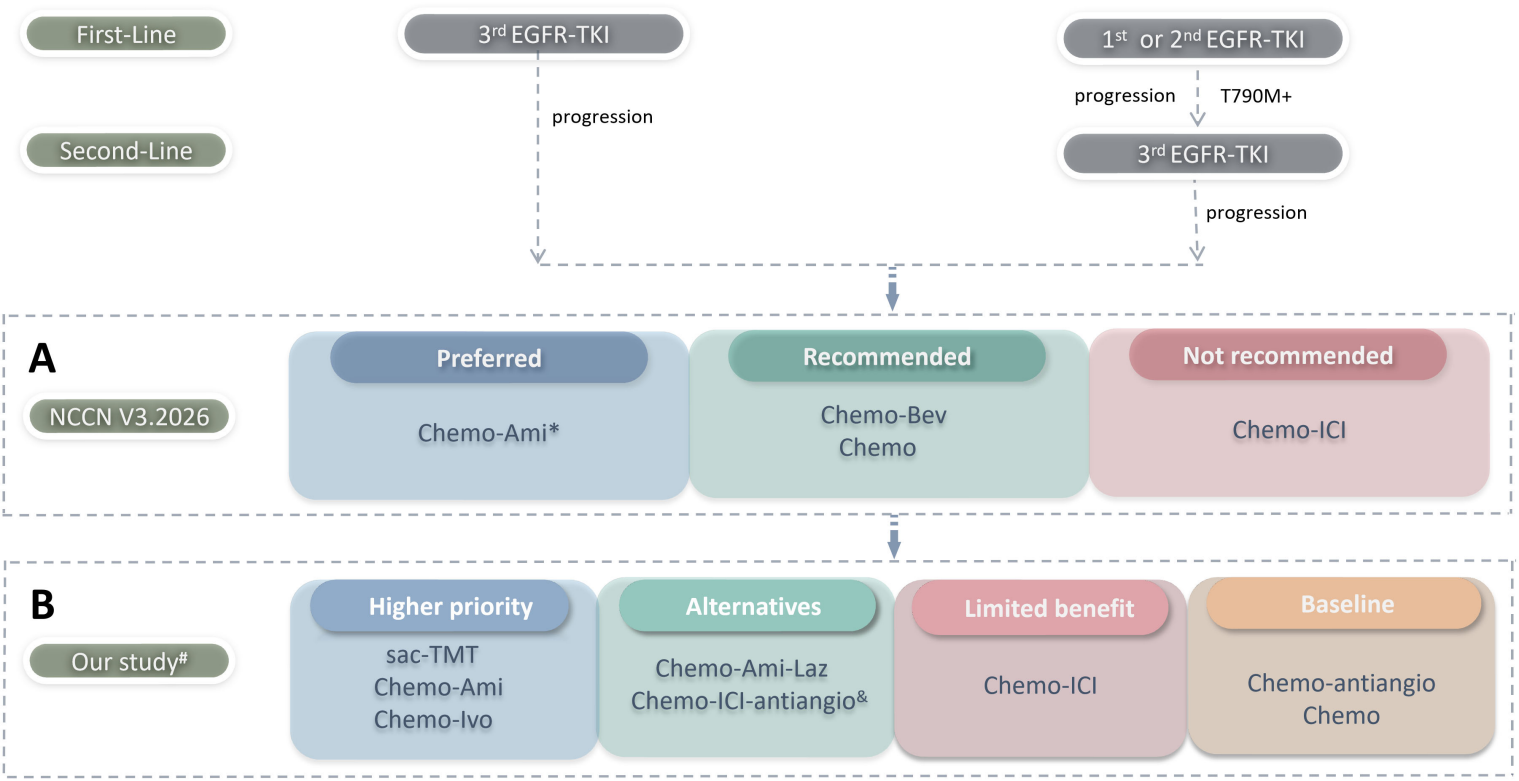
New treatment options for EGFR-mutant NSCLC. **(A)** NCCN Clinical Practice Guidelines in Oncology for Non–Small Cell Lung Cancer, Version 3.2026. **(B)** Proposed Treatment of Our study. This figure juxtaposes current NCCN guideline recommendations with an evidence-informed treatment prioritization framework derived from Bayesian network meta-analysis and probabilistic multi-criteria decision analysis (pMCDA). The framework is intended to support clinical interpretation rather than to replace guideline-level recommendations. *not an option for EGFR S768l, L861Q, G719X mutations. ^#^Based on Bayesian network meta-analysis and probabilistic multi-criteria decision analysis (probabilistic multi-criteria decision analysis was used as a decision-support tool to facilitate scenario-specific prioritization, rather than to override efficacy estimates from network meta-analysis). ^&^Chemo-ICI-antiangio is classified as an alternative option based on its integrated benefit–risk profile, but carries a low GRADE certainty of evidence due to within-study bias. Chemo, platinum-based doublet chemotherapy. ICI, immune checkpoint inhibitor; Chemo_ICI, chemotherapy plus ICI; Chemo_Antiangio, chemotherapy plus antiangiogenesis; Chemo_ICI_antiangio, chemotherapy plus antiangiogenesis plus ICI; Chemo_Ivo, chemotherapy plus Ivonescimab; sacTMT, Sacituzumab Tirumotecan; Chemo_Ami, chemotherapy plus Amivantamab; Chemo_Ami_Laz, chemotherapy plus Amivantamab plus lazertinib; TKI, tyrosine-kinase inhibitor.

To further facilitate clinical interpretation, we translated the results of the Bayesian network meta-analysis and pMCDA into an evidence-informed treatment prioritization framework and juxtaposed it with current NCCN guideline recommendations (**Figure 5**). This framework is intended to support clinical interpretation and does not constitute guideline-level recommendations. While the NCCN guidelines prioritize chemotherapy plus amivantamab and discourage ICI-containing regimens after EGFR-TKI resistance (18), our integrated analysis highlighted a broader spectrum of active treatment options and identified sac-TMT, Chemo_Ami, and Chemo_Ivo as consistently high-priority strategies based on overall benefit–risk profiles. Importantly, regimens such as Chemo_Ami_Laz or Chemo_ICI_antiangio remained evidence-supported alternatives, whereas Chemo_ICI demonstrated limited incremental benefit in this setting. Crucially, our GRADE certainty assessment (**Supplementary Figure 10**) provides strong methodological backing for these recommendations, with sac-TMT, Chemo_Ivo, amivantamab combinations consistently achieving high certainty ratings across the outcomes. Conversely, the clinical utility of Chemo-ICI-antiangio may be approached with caution, as its evidence was systematically downgraded to low certainty due to within-study bias. Collectively, this comparison illustrates how integrative evidence synthesis may complement existing guidelines by supporting structured, evidence-informed treatment prioritization, while acknowledging that final treatment decisions should remain individualized.

### 4.6 Limitations

Several limitations of this study should be acknowledged. First, despite the inclusion of randomized controlled trials only, indirect comparisons are inherent to network meta-analyses. The included protocols naturally featured notable clinical heterogeneity (such as different chemotherapy backbones and prior TKI exposures), which led to some discrepancies between the fixed-effect and random-effects models in several direct comparisons. Therefore, residual heterogeneity in subsequent therapies and patient characteristics across trials may have influenced treatment effects. Second, overall survival data were immature or unavailable in some studies (16, 21, 31), which may have limited the ability to fully characterize long-term benefit for certain regimens. Third, although probabilistic multi-criteria decision analysis provides a structured approach to integrating multiple clinical endpoints, the selection and weighting of criteria inevitably involve subjective considerations and may vary across clinical contexts. In addition, a small proportion of biologically distinct patient populations, such as those with ALK rearrangements or EGFR-positive disease without prior TKI exposure, were included in some trials (29–32, 41), potentially contributing to residual clinical heterogeneity. Furthermore, for some trials (19), grade ≥3 treatment-related adverse events were not explicitly reported, and treatment-emergent adverse events were therefore used as a proxy for safety assessment, which may have affected the comparability of toxicity estimates across regimens. Finally, some treatment strategies of clinical interest, such as datopotamab deruxtecan, could not be incorporated owing to methodological incompatibility with the platinum-based chemotherapy backbone required for network construction.

## 5 Conclusion

In summary, several treatment regimens offer meaningful benefit after EGFR-TKI resistance, with clinically relevant differences emerging when survival and toxicity are jointly considered. Integrating efficacy and safety through pMCDA identified sac-TMT, Chemo_Ami, and Chemo_Ivo as regimens with relative statistical advantages in overall performance, while other strategies remain potential alternatives for specific clinical scenarios. However, these findings must be interpreted with caution as they are constrained by varying data certainties across the interconnected network; for instance, combinations such as Chemo_ICI_antiangio are supported only by a low certainty of evidence. Overall, while these comparative cross-trial estimates offer valuable insights in the absence of head-to-head trials, clinical deployment should strictly reflect the underlying strength of the evidence and individual patient risk profiles.

## Data Availability

The original contributions presented in the study are included in the article/**Supplementary Material**. Further inquiries can be directed to the corresponding author.
