# Supplementary material for: Treatment prioritization of chemotherapy-anchored regimens after EGFR-TKI failure in EGFR-mutant NSCLC: a systematic review, pairwise meta-analysis and Bayesian network meta-analysis with probabilistic multi-criteria decision analysis
Source: Front Immunol. 2026 Jul 31;17:1864144. doi: 10.3389/fimmu.2026.1864144 (PMC13473156; doi:10.3389/fimmu.2026.1864144)
Supplement: Supplementary file 1 [file Supplementaryfile1.pdf]

Supplementary Materials

Lists

Title	Content	Page
Table S1	Checklist of the PRISMA extension for network meta-analysis.	2-5
Table S2	Literature search strategy.	6-7
Table S3	Baseline characteristics of included studies.	8
Table S4	Global heterogeneity estimates across clinical outcomes.	9
Table S5	Local inconsistency (node-splitting) analysis.	10
Table S6	Comparison of model fit across consistency, inconsistency, and effect assumptions based on DIC values.	11
Table S7	Rank probability matrices for all outcomes. A) Progression-free survival. B) Overall survival. C) Objective response rate. D) Disease control rate. E) Grade⩾3 treatment-grade adverse events.	12-13
Table S8	Weighting Schemes for probabilistic Multi-Criteria Decision Analysis Composite Scores.	14
Table S9	Composite Scores Based on probabilistic Multi-Criteria Decision Analysis with Different Weighting Schemes.	15
Figure S1	Risk of bias assessment according to the Cochrane Risk of Bias Tool. A) Detailed risk of bias assessment. B) Risk of bias summary.	16
Figure S2	Pairwise meta-analysis estimates of survival outcomes using both fixed-effect and random-effects models for head-to-head comparisons versus chemotherapy. A) Progression-free survival. B) Overall survival.	17-18
Figure S3	Convergence diagnostics of the Bayesian network meta-analysis for progression-free survival.	19
Figure S4	Comparison-adjusted funnel plot for progression-free survival.	20
Figure S5	League Table for the network meta-analysis. A) Progression-free survival and Overall survival. B) Objective response rate and Disease control rate. C) Grade⩾3 treatment-grade adverse events.	21
Figure S6	Efficacy and Safety Rankings Based on Cumulative Ranking Probabilities. A) Progression-free survival. B) Overall survival. C) Objective response rate. D) Grade⩾3 treatment-grade adverse events.	22
Figure S7	Efficacy and safety rankings Based on SUCRA. A) Progression-free survival. B) Overall survival. C) Objective response rate. D) Grade⩾3 treatment-grade adverse events.	23
Figure S8	Sensitivity analysis for grade ⩾3 TRAEs excluding the TEAE-reporting trial (HARMONi-A). A) Forest plot. B) SUCRA.	24
Figure S9	League tables of key safety endpoints. A) Serious adverse event. B) Treatment-related deaths. C) Treatment discontinuation. D) Anemia. E) Decreased WBC count. F) Decreased platelet count.	25-26
Figure S10	GRADE certainty of evidence. A) Progression-free survival. B) Overall survival. C) Objective response rate. D) Disease control rate. E) Grade⩾3 treatment-grade adverse events.	27
References	References	28

Table S1. Checklist of the PRISMA extension for network meta-analysis.

Section/topic	#	Checklist item*	Reported Section #
**TITLE**			
Title	1	Identify the report as a systematic review *incorporating a network meta-analysis (or related form of meta-analysis).*	Title
**ABSTRACT**			
Structured summary	2	Provide a structured summary including, as applicable:<br>•Background: main objectives;<br>•Methods: data sources; study eligibility criteria, participants, and interventions; study appraisal and *synthesis methods, such as network metaanalysis.*<br>•Results: number of studies and participants identified; summary estimates with corresponding confidence/credible intervals; *treatment rankings may also be discussed. Authors may choose to summarize pairwise comparisons against a chosen treatment included in their analyses for brevity.*<br>•Discussion/Conclusions: limitations; conclusions and implications of findings.<br>•Other: primary source of funding; systematic review registration number with registry name.	Abstract
**INTRODUCTION**			
Rationale	3	Describe the rationale for the review in the context of what is already known, *including mention of why a network meta-analysis has been conducted.*	Introduction
Objectives	4	Provide an explicit statement of questions being addressed with reference to participants, interventions, comparisons, outcomes, and study design (PICOS).	Introduction
**METHODS**			
Protocol and registration	5	Indicate if a review protocol exists, if and where it can be accessed (e.g., Web address), and, if available, provide registration information including registration number.	Methods
Eligibility criteria	6	Specify study characteristics (e.g., PICOS, length of follow-up) and report characteristics (e.g., years considered, language, publication status) used as criteria for eligibility, giving rationale. *Clearly describe eligible treatments included in the treatment network, and note whether any have been clustered or merged into the same node (with justification).*	Methods - Eligibility criteria
Information sources	7	Describe all information sources (e.g., databases with dates of coverage, contact with study authors to identify additional studies) in the search and date last searched.	Methods - Literature searching strategy
Search	8	Present full electronic search strategy for at least one database, including any limits used, such that it could be repeated.	Supplemental pp 6-7

Study selection	9	State the process for selecting studies (i.e., screening, eligibility, included in systematic review, and, if applicable, included in the meta-analysis).	Methods - Eligibility criteria
Data collection process	10	Describe method of data extraction from reports (e.g., piloted forms, independently, in duplicate) and any processes for obtaining and confirming data from investigators.	Methods - Data extraction and quality assessment
Data items	11	List and define all variables for which data were sought (e.g., PICOS, funding sources) and any assumptions and simplifications made.	Methods - Literature searching strategy
Geometry of the network	S1	Describe methods used to explore the geometry of the treatment network under study and potential biases related to it. This should include how the evidence base has been graphically summarized for presentation, and what characteristics were compiled and used to describe the evidence base to readers.	Methods - Statistical analysis
Risk of bias in individual studies	12	Describe methods used for assessing risk of bias of individual studies (including specification of whether this was done at the study or outcome level), and how this information is to be used in any data synthesis.	Methods - Statistical analysis
Summary measures	13	State the principal summary measures (e.g., risk ratio, difference in means). *Also describe the use of additional summary measures assessed, such as treatment rankings and surface under the cumulative ranking curve (SUCRA) values, as well as modified approaches used to present summary findings from meta-analyses.*	Methods - Statistical analysis
Synthesis of results	14	Describe the methods of handling data and combining results of studies for each network meta-analysis. This should include, but not be limited to:<br>• *Handling of multi-arm trials;*<br>• *Selection of variance structure;*<br>• *Selection of prior distributions in Bayesian analyses; and*<br>• *Assessment of model fit.*	Methods - Statistical analysis
Assessment of Inconsistency	S2	Describe the statistical methods used to evaluate the agreement of direct and indirect evidence in the treatment network(s) studied. Describe efforts taken to address its presence when found.	Methods - Statistical analysis
Risk of bias across studies	15	Specify any assessment of risk of bias that may affect the cumulative evidence (e.g., publication bias, selective reporting within studies).	Methods - Statistical analysis
Additional analyses	16	Describe methods of additional analyses, if done, indicating which were prespecified. This may include, but not be limited to the following:<br>• Sensitivity or subgroup analyses;<br>• Meta-regression analyses;	Methods - Statistical analysis

		• *Alternative formulations of the treatment network; and*<br>• *Use of alternative prior distributions for Bayesian analyses (if applicable).*	
**RESULTS**			
Study selection	17	Give numbers of studies screened, assessed for eligibility, and included in the review, with reasons for exclusions at each stage, ideally with a flow diagram.	Results- PRISMA study search results
Presentation of network structure	S3	Provide a network graph of the included studies to enable visualization of the geometry of the treatment network	Results- Efficacy and safety outcomes
Summary of network geometry	S4	Provide a brief overview of characteristics of the treatment network. This may include commentary on the abundance of trials and randomised patients for the different interventions and pairwise comparisons in the network, gaps of evidence in the treatment network, and potential biases reflected by the network structure.	Results- Study and population characteristics, Risk of bias assessment, Network topology and quality assessment
Study characteristics	18	For each study, present characteristics for which data were extracted (e.g., study size, PICOS, follow-up period) and provide the citations.	Supplemental p8
Risk of bias within studies	19	Present data on risk of bias of each study and, if available, any outcome level assessment.	Supplemental p16
Results of individual studies	20	For all outcomes considered (benefits or harms), present, for each study:1) simple summary data for each intervention group, and 2) effect estimates and confidence/credible intervals. *Modified approaches may be needed to deal with information from larger networks.*	Results- Efficacy and safety outcomes
Synthesis of results	21	Present results of each meta-analysis done, including confidence/credible intervals. *In larger networks, authors may focus on comparisons versus a particular comparator (e.g. placebo or standard care), with full findings presented in an appendix. League tables and forest plots may be considered to summarize pairwise comparisons.* If additional summary measures were explored (such as treatment rankings), these should also be presented.	Results- Efficacy and safety outcomes , Supplemental pp 21-26
Exploration for inconsistency	S5	Describe results from investigations of inconsistency. This may include such information as measures of model fit to compare consistency and inconsistency models, P values from statistical tests, or summary of inconsistency estimates from different parts of the treatment network.	Results- Network topology and quality assessment
Risk of bias across studies	22	Present results of any assessment of risk of bias across studies.	Results- Risk of bias

			assessment
Additional analysis	23	Give results of additional analyses, if done (e.g., sensitivity or subgroup analyses, meta-regression, *alternative network geometries studied, alternative choice of prior distributions for Bayesian analyses,* and so forth).	Results-Network topology and quality assessment
**DISCUSSION**			
Summary of evidence	24	Summarize the main findings including the strength of evidence for each main outcome; consider their relevance to key groups (e.g., healthcare providers, users, and policy makers).	Discussion
Limitations	25	Discuss limitations at study and outcome level (e.g., risk of bias), and at review-level (e.g., incomplete retrieval of identified research, reporting bias). *Comment on the validity of the assumptions, such as transitivity and consistency. Comment on any concerns regarding network geometry (e.g., avoidance of certain comparisons).*	Limitations
Conclusions	26	Provide a general interpretation of the results in the context of other evidence, and implications for future research.	Conclusions
**FUNDING**			
Funding	27	Describe sources of funding for the systematic review and other support (e.g., supply of data); role of funders for the systematic review.	Funding

PRISMA = Preferred Reporting Items for Systematic Reviews and Meta-Analysis; PICOS = population, intervention, comparators, outcomes, study design.

*Text in italics indicates wording specific to reporting of network meta-analyses that has been added to guidance from the PRISMA statement.

Table S2. Literature search strategy.

ID	Search strategies	Hits
**PubMed**		
#1	"carcinoma, non small cell lung"[MeSH Terms] OR "non small cell lung cancer"[Title/Abstract] OR "NSCLC"[Title/Abstract]	118,787
#2	"epidermal growth factor receptor"[All Fields] OR "EGFR"[All Fields]	127,446
#3	"tyrosine kinase inhibitor"[All Fields] OR "kinase inhibitor"[All Fields] OR "TKI"[All Fields]	85,489
#4	("pembrolizumab" [Supplementary Concept] OR "Keytruda" [Title/Abstract] OR "MK-3475" [Title/Abstract] OR "lambrolizumab" [Title/Abstract] OR "nivolumab" [Supplementary Concept] OR "Opdivo"[Title/Abstract] OR "ONO-4538" [Title/Abstract] OR "MDX-1106" [Title/Abstract] OR "BMS-936558" [Title/Abstract] OR "atezolizumab"[Supplementary Concept] OR "Tecentriq"[Title/Abstract] OR "MPDL3280A"[Title/Abstract] OR"RG7446"[Title/Abstract] OR "RG-7446"[Title/Abstract] OR "Durvalumab" [Title/Abstract] OR "Imfinzi" [Title/Abstract] OR " MEDI4736" [Title/Abstract] OR "Camrelizumab" [Title/Abstract] OR "SHR-1210" [Title/Abstract] OR " Tislelizumab" [Title/Abstract] OR "Sintilimab" [Title/Abstract] OR " IBI 308" [Title/Abstract] OR "PD-1"[Title/Abstract] OR "PD-L1"[Title/Abstract] OR "anti-PDL1"[Title/Abstract] OR "anti-PD1"[Title/Abstract] OR "Programmed Death 1"[Title/Abstract] OR "Programmed Cell Death 1 Receptor"[Title/Abstract] OR "Programmed Death Ligand 1"[Title/Abstract] OR "programmed cell death 1 ligand 1 protein"[Title/Abstract] OR "immune checkpoint inhibitor"[Title/Abstract] OR "immune therapy"[Title/Abstract] OR "immunotherapy"[Title/Abstract]) OR ("Bevacizumab"[Title/Abstract] OR "Avastin"[Title/Abstract])	248,677
#5	(("clinical trials as topic"[MeSH Terms]) OR "Randomized clinical trial"[Title/Abstract]) OR ''phase''[Title/Abstract]	1,750,799
#6	"0001/01/01"[Date - Publication] : "2025/12/19"[Date - Publication]	39,874,491
#7	#1 AND #2 AND #3 AND #4 AND #5	153
**Embase**		
#1	'non small cell lung cancer':ab,ti OR 'nsclc':ab,ti	182,681
#2	'epidermal growth factor receptor':ab,ti OR 'egfr':ab,ti	223,822
#3	'tyrosine kinase inhibitor':ab,ti OR 'kinase inhibitor':ab,ti OR 'tki':ab,ti	95,241
#4	(‘pembrolizumab’/exp OR ‘Keytruda’:ab,ti OR ‘MK-3475’:ab,ti OR ‘lambrolizumab’:ab,ti OR ‘nivolumab’/exp OR ‘Opdivo’:ab,ti OR ‘ONO-4538’:ab,ti OR ‘MDX-1106’:ab,ti OR ‘BMS-936558’:ab,ti OR ‘atezolizumab’/exp OR ‘Tecentriq’:ab,ti OR ‘MPDL3280A’:ab,ti OR ‘RG7446’:ab,ti OR ‘RG-7446’:ab,ti OR ‘Durvalumab’:ab,ti OR ‘Imfinzi’:ab,ti OR ‘MEDI4736’:ab,ti OR ‘Camrelizumab’/exp OR ‘SHR-1210’:ab,ti OR ‘Tislelizumab’/exp OR ‘Sintilimab’/exp OR ‘IBI-308’:ab,ti OR ‘PD-1’:ab,ti OR ‘PD-L1’:ab,ti OR ‘anti-PDL1’:ab,ti OR ‘anti-PD1’:ab,ti OR ‘Programmed Death 1’:ab,ti OR ‘Programmed Cell Death 1 Receptor’:ab,ti OR ‘Programmed Death-Ligand 1’:ab,ti OR ‘programmed cell death 1 ligand 1 protein’:ab,ti OR ‘immune checkpoint inhibitor’:ab,ti OR ‘immune therapy’:ab,ti OR ‘immunotherapy’:ab,ti) OR (‘Bevacizumab’:ab,ti OR ‘Avastin’:ab,ti)	419,729
#5	([clinical study]/lim OR [controlled clinical trial]/lim OR [randomized controlled trial]/lim) AND [01-01-0001]/sd NOT [19-12-2025]/sd	14,594,238
#6	#1 AND #2 AND #3 AND #4 AND #5	1,342
**Cochrane Library**		
#1	("carcinoma, non small cell lung" OR "non small cell lung cancer" OR "NSCLC"):ti,ab,kw with Cochrane Library publication date Between Jan 0001 and Dec 2025, in Trials	18864
#2	("epidermal growth factor receptor" OR "EGFR"):ti,ab,kw	19360
#3	(("tyrosine kinase inhibitor" OR "kinase inhibitor" OR "TKI") AND ("pembrolizumab" OR "Keytruda" OR "MK-3475" OR "lambrolizumab" OR "nivolumab" OR "Opdivo" OR "ONO-4538" OR "MDX-1106" OR "BMS-936558" OR "atezolizumab" OR "Tecentriq" OR "MPDL3280A" OR "RG7446" OR "RG-7446" OR "Durvalumab" OR "Imfinzi" OR "MEDI4736" OR "Camrelizumab" OR "SHR-1210" OR "Tislelizumab" OR "Sintilimab" OR "IBI 308" OR "PD-1" OR "PD-L1" OR "anti-PDL1" OR "anti-PD1" OR "Programmed Death 1" OR "Programmed Cell Death 1 Receptor" OR "Programmed Death-Ligand 1" OR "programmed	1090

ID	Search strategies	Hits
	cell death 1 ligand 1 protein" OR "immune checkpoint inhibitor" OR "immune therapy" OR "immunotherapy" OR "Bevacizumab" OR "Avastin")):ti,ab,kw	
#4	#1 AND #2 AND #3	286

Table S3. Baseline characteristics of included studies.

Trial (Phase, Year)	Registered Number	Country	No.	Regimen	Drug name	Efficacy outcomes				
						PFS HR	OS HR	ORR	DCR	≥3 TRAEs
CheckMate 722 (III, 2024[1])	NCT02864251	Global	144	Chemo-ICI	Nivolumab + Carboplatin/cisplatin-pemetrexed	√	√	√	√	√[g]
			150	Chemo	Carboplatin/cisplatin-pemetrexed					
KEYNOTE-789 (III, 2024[2])	NCT03515837	Global	245	Chemo-ICI	Pembrolizumab + Carboplatin/cisplatin-pemetrexed	√	√	√	√	√[g]
			247	Chemo	Carboplatin/cisplatin-pemetrexed					
IMpower150 (III, 2019,2021[3, 4])	NCT02366143	Global	22	Chemo-ICI-antiangio	Atezolizumab + Bevacizumab + Carboplatin-paclitaxel	√	√	√[c]	√[c]	√[d]
			28	Chemo-ICI	Atezolizumab + Carboplatin-paclitaxel					
			28	Chemo-antiangio	Bevacizumab + Carboplatin-paclitaxel					
ORIENT-31 (III, 2022, 2023[5, 6])	NCT03802240	China	158	Chemo-ICI-antiangio	Sintilimab + IBI-305 + Cisplatin-pemetrexed	√	√	√	√	√[g]
			158	Chemo-ICI	Sintilimab + Cisplatin-pemetrexed					
			160	Chemo	Cisplatin-pemetrexed					
ATTLAS (III, 2023[7])	NCT03991403	Korea	144	Chemo-ICI-antiangio	Atezolizumab + Bevacizumab + Carboplatin-paclitaxel	√	√	√[a]	√[a]	√[a]
			68	Chemo	Pemetrexed-carboplatin/cisplatin					
IMpower151 (III, 2025[8])	NCT04194203	China	79	Chemo-ICI-antiangio	Atezolizumab + Bevacizumab + Carboplatin-pemetrexed/paclitaxel	√[b]	√[b,i]	√[b]		
			79	Chemo-antiangio	Bevacizumab + Carboplatin-pemetrexed/paclitaxel					
MARIPOSA-2 (III, 2023, 2024[9, 10])	NCT04988295	Global	131	Chemo-Ami	Amivantamab + Pemetrexed-Carboplatin	√	√[i]	√	√	√[g]
			263	Chemo-Ami-Laz	Amivantamab + lazertinib+ Pemetrexed-Carboplatin					
			263	Chemo	Pemetrexed-Carboplatin					
HARMONi-A (III, 2024, 2025[11, 12])	NCT05184712	China	161	Chemo-Ivo	Ivonescimab + Pemetrexed-Carboplatin	√	√	√	√	√[h]
			161	Chemo	Pemetrexed-Carboplatin					
APPLE(WJOG11218L) (III, 2024[13])	jRCT2080224500	Japan	51	Chemo-ICI-antiangio	Atezolizumab + Bevacizumab + Pemetrexed-Carboplatin	√	√	√[e]		
			52	Chemo-ICI	Atezolizumab + Pemetrexed-Carboplatin					
OptiTROP-Lung04 (III, 2025[14])	NCT05870319	China	188	sac-TMT	sac-TMT	√	√	√	√	√[g]
			188	Chemo	Pemetrexed-Carboplatin/cisplatin					
HARMONi (III, 2025[15])	NCT06396065	Global	219	Chemo-Ivo	Ivonescimab + Pemetrexed-Carboplatin	√[f]	√[i]	√	√	√[g]
			219	Chemo	Pemetrexed-Carboplatin					

[a] Includes 13 patients with ALK rearrangement. [b] Includes 5 patients with ALK rearrangement. [c] Includes 44 EGFR+ TKI-naïve patients. [d] Includes 35 EGFR+ TKI-naïve patients. [e] Includes 14 driver oncogene–positive patients who were TKI-naïve or without EGFR mutations. [f] n = 172 vs 173. [g] Excludes patients who did not receive treatment. [h] TEAEs. [i] Not mature (IMpower 151; Ami-Laz-Chemo vs. Chemo in MARIPOSA-2; western population in HARMONI).

Chemo= platinum-based doublet chemotherapy. ICI=immune checkpoint inhibitor. Chemo_ICI=chemotherapy plus ICI. Chemo_Antiangio=chemotherapy plus antiangiogenesis. Chemo_ICI_antiangio=chemotherapy plus antiangiogenesis plus ICI. Chemo_Ivo=chemotherapy plus Ivonescimab. sacTMT= Sacituzumab Tirumotecan. Chemo_Ami=chemotherapy plus Amivantamab. Chemo_Ami_Laz=chemotherapy plus Amivantamab plus lazertinib. PFS=Progression-free survival. OS=Overall survival. ORR=Objective response rate and disease control rate. DCR= Disease control rate. ≥3 TRAEs =Grade⩾3 treatment-grade adverse events. HR=hazard ratio.

Table S4. Global heterogeneity estimates across clinical outcomes.

	I^2	sd.d	95% CrI	
**PFS**	0%	0.086	0.003	0.289
**OS**	0%	0.093	0.003	0.298
**ORR**	10%	0.239	0.008	0.748
**DCR**	0%	0.216	0.007	0.723
**≥3 TRAEs**	7%	0.442	0.053	1.196

PFS=Progression-free survival. OS=Overall survival. ORR=Objective response rate and disease control rate. DCR= Disease control rate. ≥3 TRAEs =Grade≥3 treatment-grade adverse events. CrI=Credible Interval.

Table S5. Local inconsistency (node-splitting) analysis.

Nodes	Log (HR/OR) with 95% CrI			p
	Direct	Indirect	Overall	
**PFS**				
Chemo_ICI vs. Chemo	-0.27 (-0.50, -0.06)	-0.17 (-0.65, 0.35)	-0.26 (-0.43, -0.08)	0.692
Chemo_ICI_antiangio vs. Chemo	-0.59 (-0.87, -0.30)	-0.69 (-1.2, -0.24)	-0.61 (-0.84, -0.39)	0.688
Chemo_ICI_antiangio vs. Chemo_ICI	-0.36 (-0.85, 0.14)	-0.36 (-0.69, -0.05)	-0.35 (-0.61, -0.10)	0.996
Chemo_antiangio vs. Chemo_ICI	-0.22 (-0.84, 0.39)	0.001 (-0.44, 0.50)	-0.07 (-0.43, 0.28)	0.563
Chemo_ICI_antiangio vs. Chemo_antiangio	-0.33 (-0.73, 0.01)	-0.12 (-0.79, 0.55)	-0.28 (-0.60, 0.02)	0.571
**OS**				
Chemo_ICI vs. Chemo	-0.15 (-0.33, 0.05)	0.37 (-0.18, 0.90)	-0.10 (-0.27, 0.13)	0.082
Chemo_ICI_antiangio vs. Chemo	-0.01 (-0.30, 0.27)	-0.52 (-1.0, -0.03)	-0.14 (-0.41, 0.11)	0.078
Chemo_ICI_antiangio vs. Chemo_ICI	-0.33 (-0.92, 0.26)	0.04 (-0.31, 0.34)	-0.05 (-0.36, 0.22)	0.278
Chemo_antiangio vs. Chemo_ICI	-0.20 (-0.82, 0.44)	0.27 (-0.24, 0.77)	0.09 (-0.33, 0.49)	0.245
Chemo_ICI_antiangio vs. Chemo_antiangio	-0.27 (-0.68, 0.16)	0.21 (-0.50, 0.90)	-0.14 (-0.50, 0.21)	0.245
**ORR**				
Chemo_ICI vs. Chemo	0.18 (-0.44, 0.80)	0.60 (-0.86, 1.90)	0.23 (-0.17, 0.65)	0.495
Chemo_ICI_antiangio vs. Chemo	0.96 (0.17, 1.80)	0.71 (-0.39, 1.90)	0.87 (0.42, 1.40)	0.672
Chemo_ICI_antiangio vs. Chemo_ICI	0.64 (0.09, 1.30)	1.00 (-0.18, 2.20)	0.65 (0.22, 1.10)	0.493
Chemo_antiangio vs. Chemo_ICI	0.27 (-0.72, 1.20)	0.93 (0.04, 1.80)	0.51 (-0.25, 1.20)	0.311
**DCR**				
Chemo_ICI vs. Chemo	0.37 (-0.13, 0.93)	1.00 (-1.00, 3.10)	0.39 (-0.01, 0.86)	0.529
Chemo_ICI_antiangio vs. Chemo	0.85 (0.06, 1.80)	0.66 (-1.00, 2.40)	0.80 (0.22, 1.40)	0.826
Chemo_ICI_antiangio vs. Chemo_ICI	0.33 (-0.50, 1.20)	0.90 (-0.72, 2.60)	0.41 (-0.18, 1.00)	0.504
**≥3 TRAEs**				
Chemo_ICI vs. Chemo	0.17 (-0.79, 1.10)	1.00 (-1.50, 3.50)	0.24 (-0.35, 0.90)	0.403
Chemo_ICI_antiangio vs. Chemo	0.60 (-0.36, 1.80)	0.52 (-1.50, 2.50)	0.67 (-0.04, 1.40)	0.916
Chemo_ICI_antiangio vs. Chemo_ICI	0.47 (-0.52, 1.30)	0.75 (-0.76, 2.20)	0.43 (-0.33, 1.20)	0.647

Chemo= platinum-based doublet chemotherapy. ICI=immune checkpoint inhibitor. Chemo_ICI=chemotherapy plus ICI. Chemo_Antiangio=chemotherapy plus antiangiogenesis. Chemo_ICI_antiangio=chemotherapy plus antiangiogenesis plus ICI. Chemo_Ivo=chemotherapy plus Ivonescimab. sacTMT= Sacituzumab Tirumotecan. Chemo_Ami=chemotherapy plus Amivantamab. Chemo_Ami_Laz=chemotherapy plus Amivantamab plus lazertinib. PFS=Progression-free survival. OS=Overall survival. ORR=Objective response rate and disease control rate. DCR= Disease control rate. ≥3 TRAEs =Grade≥3 treatment-grade adverse events. HR=hazard ratio. OR= Odds ratio. CrI=Credible Interval.

Table S6. Comparison of model fit across consistency, inconsistency, and effect assumptions based on DIC values.

	Model		
	**Inconsistency Random**	**Consistency Random**	**Consistency Fixed**
**PFS**	24.898	21.425	19.741
**OS**	20.713	20.663	18.779
**ORR**	47.536	47.633	46.713
**DCR**	38.440	36.660	34.879
**≥3 TRAEs**	42.227	41.521	44.412

The deviance information criterion (DIC) was used to compare model fit while accounting for model complexity, with lower values indicating a better balance between fit and parsimony; differences of 5 or more were considered meaningful.

PFS=Progression-free survival. OS=Overall survival. ORR=Objective response rate and disease control rate. DCR= Disease control rate. ≥3 TRAEs =Grade≥3 treatment-grade adverse events.

Table S7. Rank probability matrices for all outcomes. A) Progression-free survival. B) Overall survival. C) Objective response rate. D) Disease control rate. E) Grade⩾3 treatment-grade adverse events.

A. PFS

	1	2	3	4	5	6	7	8
Chemo	0.00000	0.00000	0.00000	0.00005	0.00040	0.00315	0.03700	0.95940
Chemo_Ami	0.24355	0.22065	0.19040	0.15680	0.14320	0.03565	0.00830	0.00145
Chemo_Ami_laz	0.45120	0.24555	0.14670	0.09360	0.04980	0.01040	0.00215	0.00060
Chemo_antiangio	0.00205	0.00580	0.01155	0.02685	0.07295	0.55695	0.29250	0.03135
Chemo_ICI	0.00010	0.00015	0.00090	0.00345	0.01700	0.32090	0.65175	0.00575
Chemo_ICI_antiangio	0.02635	0.08850	0.16975	0.27850	0.41950	0.01690	0.00045	0.00005
Chemo_Ivo	0.11545	0.22135	0.26080	0.23740	0.14095	0.02140	0.00225	0.00040
sacTMT	0.16130	0.21800	0.21990	0.20335	0.15620	0.03465	0.00560	0.00100

Rank 1 indicates the best treatment.

B. OS

	1	2	3	4	5	6	7	8
Chemo	0.00000	0.00020	0.00300	0.02035	0.09270	0.26220	0.40055	0.22100
Chemo_Ami	0.18770	0.34275	0.19620	0.10680	0.06725	0.04335	0.03260	0.02335
Chemo_Ami_laz	0.02405	0.06425	0.09290	0.12660	0.11235	0.12570	0.15750	0.29665
Chemo_antiangio	0.01100	0.03645	0.06095	0.09260	0.11410	0.13585	0.16730	0.38175
Chemo_ICI	0.00125	0.01470	0.06830	0.21270	0.28560	0.24000	0.13485	0.04260
Chemo_ICI_antiangio	0.01335	0.06970	0.16280	0.25590	0.23735	0.14850	0.08735	0.02505
Chemo_Ivo	0.07095	0.29040	0.34820	0.15705	0.07605	0.03700	0.01485	0.00550
sacTMT	0.69170	0.18155	0.06765	0.02800	0.01460	0.00740	0.00500	0.00410

Rank 1 indicates the best treatment.

C. ORR

	1	2	3	4	5	6	7	8
Chemo	0.00000	0.00006	0.00019	0.00129	0.00521	0.02424	0.12910	0.83991
Chemo_Ami	0.44871	0.27576	0.12545	0.07664	0.04329	0.01868	0.00725	0.00423
Chemo_Ami_laz	0.30314	0.35806	0.15480	0.09843	0.05053	0.02173	0.00874	0.00459
Chemo_antiangio	0.08075	0.09554	0.15150	0.22088	0.21668	0.16884	0.04269	0.02314
Chemo_ICI	0.00020	0.00104	0.00450	0.01476	0.05150	0.18291	0.65866	0.08643
Chemo_ICI_antiangio	0.08418	0.15849	0.33829	0.27501	0.11840	0.02414	0.00126	0.00024
Chemo_Ivo	0.01084	0.02331	0.06010	0.12445	0.25855	0.40275	0.10118	0.01883
sacTMT	0.07219	0.08774	0.16518	0.18855	0.25585	0.15673	0.05113	0.02265

Rank 1 indicates the best treatment.

D. DCR

	1	2	3	4	5	6	7	8
Chemo	0.00000	0.00001	0.00018	0.00089	0.00460	0.02253	0.17488	0.79693
Chemo_Ami	0.39434	0.27333	0.15131	0.08661	0.05156	0.02565	0.01275	0.00445

Chemo_Ami_laz	0.23519	0.31275	0.21303	0.11964	0.06739	0.03154	0.01505	0.00543
Chemo_antiangio	0.21431	0.11665	0.12054	0.11380	0.12385	0.11346	0.10270	0.09469
Chemo_ICI	0.00089	0.00506	0.01586	0.05034	0.15675	0.37080	0.38451	0.01579
Chemo_ICI_antiangio	0.06838	0.13043	0.21666	0.25869	0.20228	0.09446	0.02621	0.00290
Chemo_Ivo	0.05443	0.11186	0.19825	0.23528	0.21831	0.12458	0.05128	0.00603
sacTMT	0.03248	0.04991	0.08418	0.13476	0.17526	0.21699	0.23263	0.07380

Rank 1 indicates the best treatment.

E. Grade⩾3 TRAEs

	1	2	3	4	5	6	7	8
Chemo	0.34813	0.39935	0.18294	0.05446	0.01269	0.00214	0.00028	0.00003
Chemo_Ami	0.00739	0.00916	0.01398	0.02308	0.04588	0.10429	0.77546	0.02078
Chemo_Ami_laz	0.00040	0.00075	0.00100	0.00163	0.00329	0.00634	0.02983	0.95678
Chemo_antiangio	0.26579	0.11788	0.12749	0.13818	0.15675	0.13508	0.05133	0.00753
Chemo_ICI	0.05843	0.17603	0.30795	0.28440	0.13276	0.03415	0.00571	0.00058
Chemo_ICI_antiangio	0.00586	0.01801	0.04468	0.11224	0.26534	0.46661	0.08148	0.00579
Chemo_Ivo	0.05338	0.09591	0.15859	0.23205	0.26306	0.16303	0.03043	0.00356
sacTMT	0.26064	0.18291	0.16339	0.15398	0.12024	0.08838	0.02550	0.00498

Rank 1 indicates the lowest risk.

Chemo= platinum-based doublet chemotherapy. ICI=immune checkpoint inhibitor. Chemo_ICI=chemotherapy plus ICI. Chemo_Antiangio=chemotherapy plus antiangiogenesis. Chemo_ICI_antiangio=chemotherapy plus antiangiogenesis plus ICI. Chemo_Ivo=chemotherapy plus Ivonescimab. sacTMT= Sacituzumab Tirumotecan. Chemo_Ami=chemotherapy plus Amivantamab. Chemo_Ami_Laz=chemotherapy plus Amivantamab plus lazertinib.

Table S8. Weighting Schemes for probabilistic Multi-Criteria Decision Analysis Composite Scores.

	PFS	OS	ORR	DCR	Grade⩾3 TRAEs
Scheme A (Survival-Focused)	0.30	0.40	0.10	0.10	0.10
Scheme B (Balanced)	0.25	0.35	0.15	0.10	0.15
Scheme C (Safety-Focused)	0.20	0.20	0.10	0.10	0.40

PFS=Progression-free survival. OS=Overall survival. ORR=Objective response rate and disease control rate. DCR= Disease control rate. ≥3 TRAEs =Grade⩾3 treatment-grade adverse events.

Table S9. Composite Scores Based on probabilistic Multi-Criteria Decision Analysis with Different Weighting Schemes.

	Scheme A (Survival-Focused)		Scheme B (Balanced)		Scheme C (Safety-Focused)	
Treatment	Mean score (95% CrI)	P(best)	Mean score (95% CrI)	P(best)	Mean score (95% CrI)	P(best)
sacTMT	0.782 (0.508–0.946)	0.541	0.771 (0.520–0.938)	0.546	0.789 (0.580–0.943)	0.609
Chemo_Ami	0.712 (0.435–0.943)	0.294	0.707 (0.452–0.925)	0.289	0.652 (0.412–0.884)	0.120
Chemo_Ivo	0.657 (0.442–0.866)	0.106	0.653 (0.454–0.847)	0.105	0.714 (0.538–0.872)	0.198
Chemo_ICI_antiangio	0.558 (0.375–0.778)	0.023	0.580 (0.406–0.782)	0.031	0.643 (0.473–0.810)	0.054
Chemo_Ami_laz	0.535 (0.359–0.830)	0.032	0.526 (0.358–0.788)	0.024	0.432 (0.291–0.631)	0.001
Chemo_antiangio	0.393 (0.188–0.684)	0.004	0.437 (0.234–0.701)	0.006	0.569 (0.332–0.772)	0.019
Chemo_ICI	0.3190 (0.182–0.484)	NA	0.348 (0.218–0.501)	NA	0.513 (0.383–0.632)	0.000
Chemo	0.100 (0.100–0.100)	NA	0.150 (0.150–0.150)	NA	0.400 (0.400–0.400)	NA

Chemo= platinum-based doublet chemotherapy. ICI=immune checkpoint inhibitor. Chemo_ICI=chemotherapy plus ICI. Chemo_Antiangio=chemotherapy plus antiangiogenesis. Chemo_ICI_antiangio=chemotherapy plus antiangiogenesis plus ICI. Chemo_Ivo=chemotherapy plus Ivonescimab. sacTMT= Sacituzumab Tirumotecan. Chemo_Ami=chemotherapy plus Amivantamab. Chemo_Ami_Laz=chemotherapy plus Amivantamab plus lazertinib. CrI= Credible Interval. P(best)=probability of being the best treatment.

Figure S1. Risk of bias assessment according to the Cochrane Risk of Bias Tool. A) Detailed risk of bias assessment. B) Risk of bias summary.

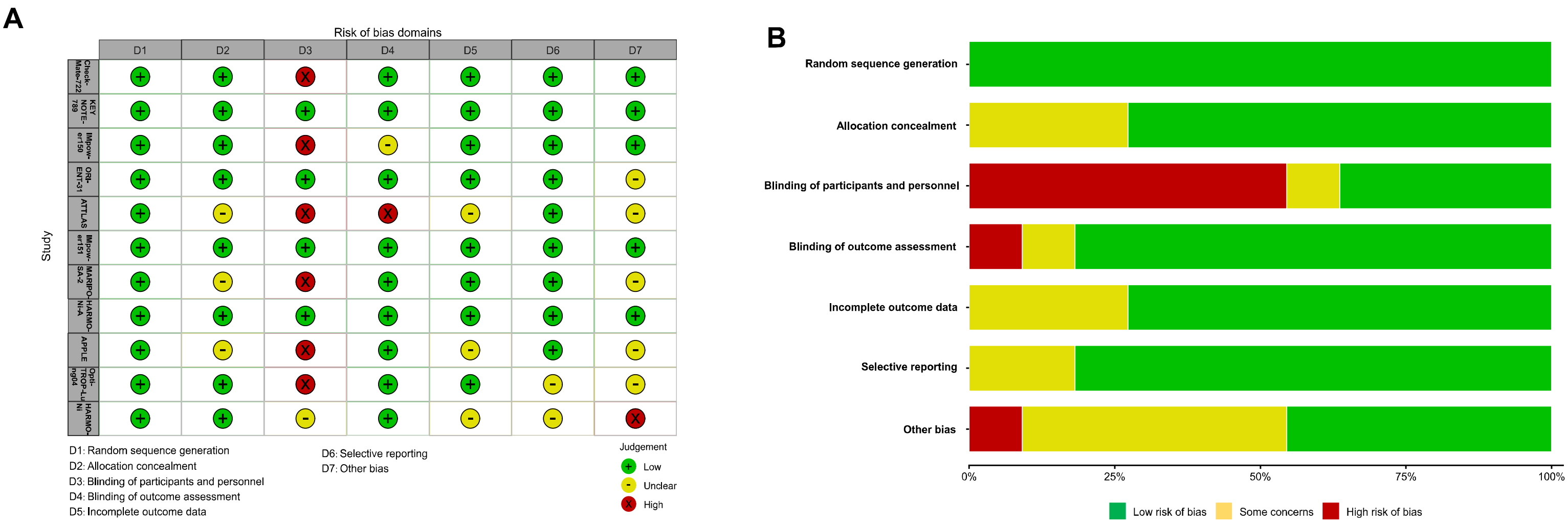

Figure S2. Pairwise meta-analysis estimates of survival outcomes using both fixed-effect and random-effects models for head-to-head comparisons versus chemotherapy. A) Progression-free survival. B) Overall survival.

Chemo= platinum-based doublet chemotherapy. ICI=immune checkpoint inhibitor. Chemo_ICI=chemotherapy plus ICI. Chemo_Antiangio=chemotherapy plus antiangiogenesis. Chemo_ICI_antiangio=chemotherapy plus antiangiogenesis plus ICI. Chemo_Ivo=chemotherapy plus Ivonescimab. sacTMT= Sacituzumab Tirumotecan. Chemo_Ami=chemotherapy plus Amivantamab. Chemo_Ami_Laz=chemotherapy plus Amivantamab plus lazertinib. CI= Confidence Interval.

A. PFS

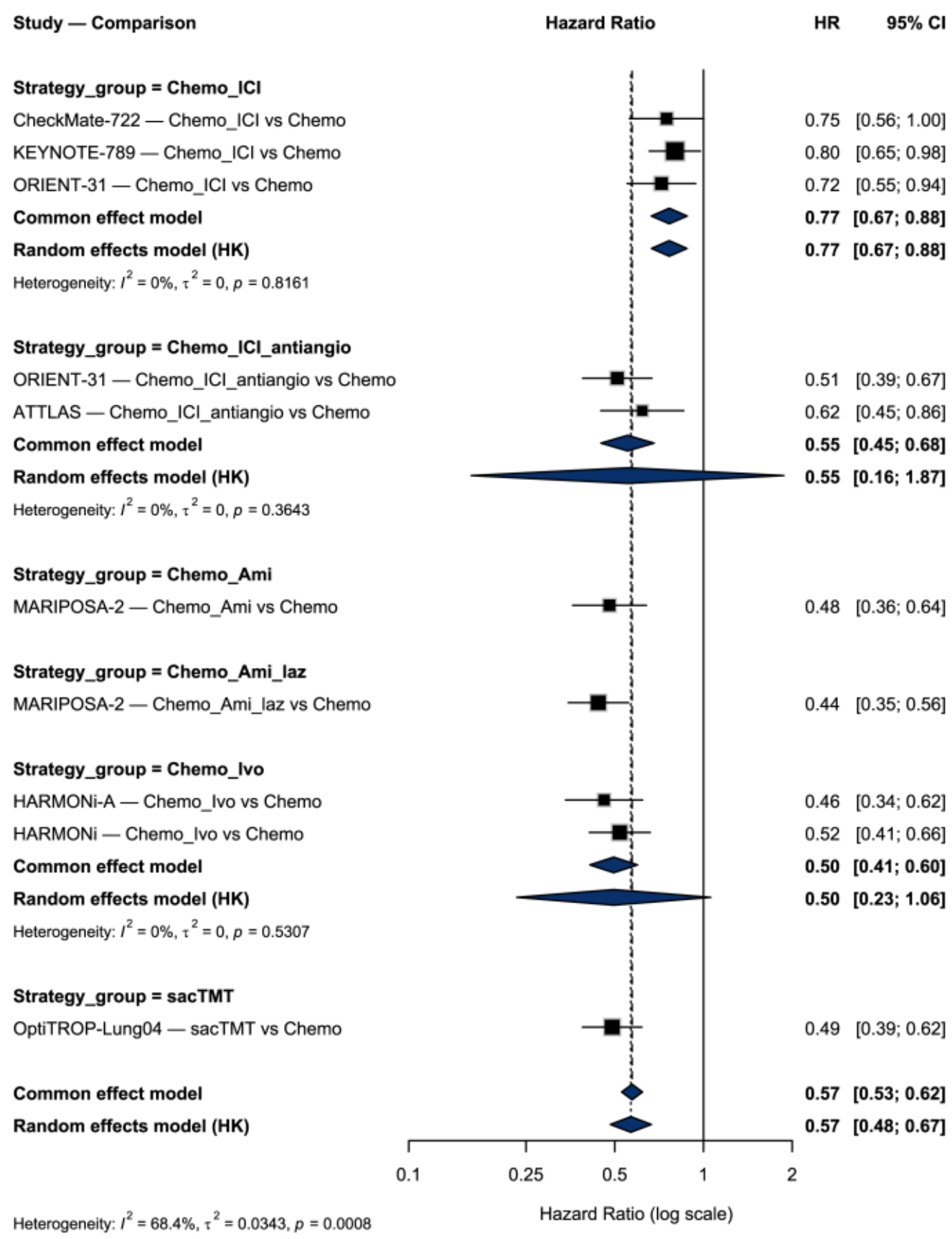

B. OS

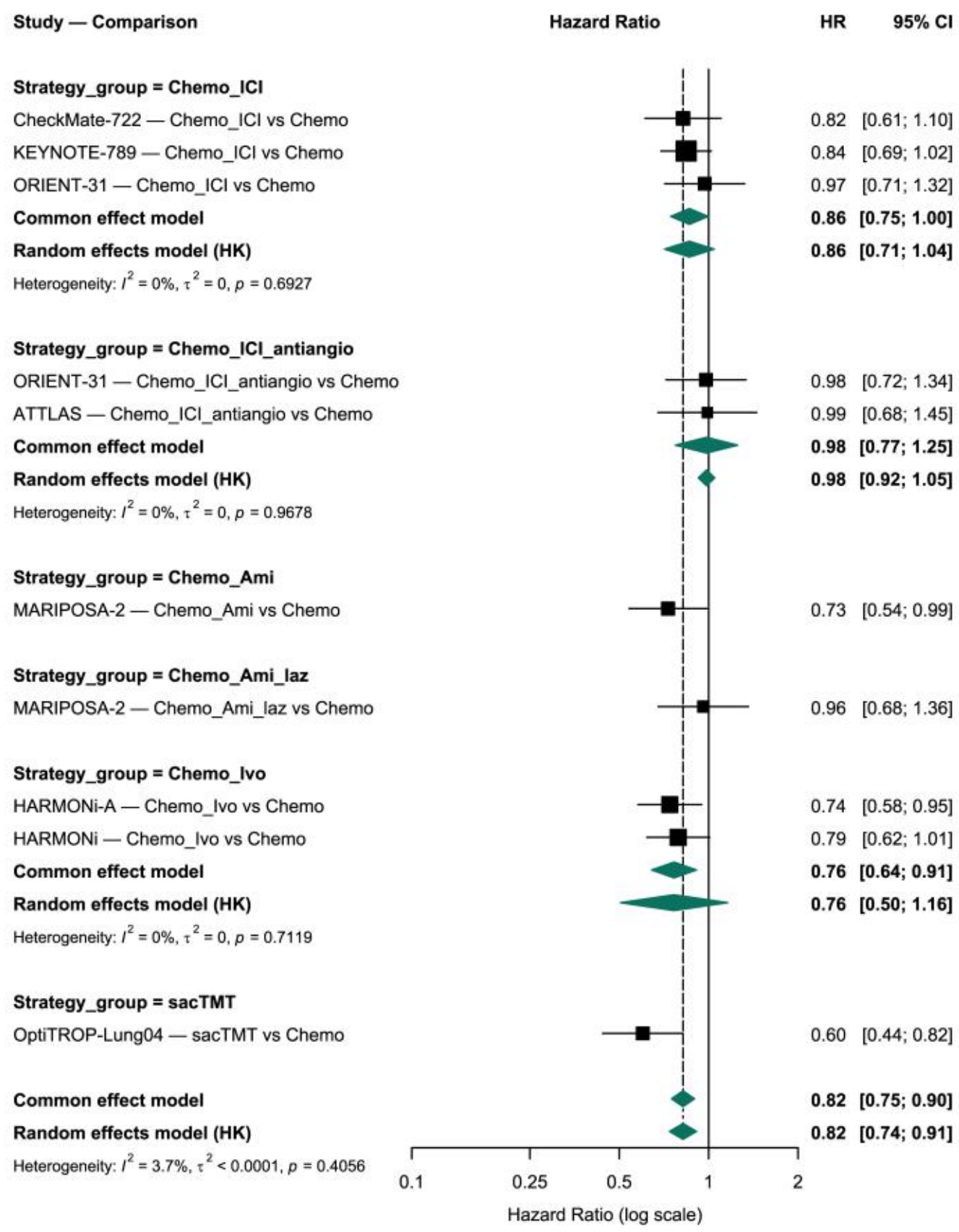

Study — Comparison
Hazard Ratio
HR
95% CI
Strategy_group = Chemo_ICI
CheckMate-722 — Chemo_ICI vs Chemo
0.82 [0.61; 1.10]
KEYNOTE-789 — Chemo_ICI vs Chemo
0.84 [0.69; 1.02]
ORIENT-31 — Chemo_ICI vs Chemo
0.97 [0.71; 1.32]
Common effect model
0.86 [0.75; 1.00]
Random effects model (HK)
0.86 [0.71; 1.04]
Heterogeneity: I^2 = 0%, τ^2 = 0, p = 0.6927
Strategy_group = Chemo_ICI_antiangio
ORIENT-31 — Chemo_ICI_antiangio vs Chemo
0.98 [0.72; 1.34]
ATTLAS — Chemo_ICI_antiangio vs Chemo
0.99 [0.68; 1.45]
Common effect model
0.98 [0.77; 1.25]
Random effects model (HK)
0.98 [0.92; 1.05]
Heterogeneity: I^2 = 0%, τ^2 = 0, p = 0.9678
Strategy_group = Chemo_Ami
MARIPOSA-2 — Chemo_Ami vs Chemo
0.73 [0.54; 0.99]
Strategy_group = Chemo_Ami_laz
MARIPOSA-2 — Chemo_Ami_laz vs Chemo
0.96 [0.68; 1.36]
Strategy_group = Chemo_Ivo
HARMONi-A — Chemo_Ivo vs Chemo
0.74 [0.58; 0.95]
HARMONi — Chemo_Ivo vs Chemo
0.79 [0.62; 1.01]
Common effect model
0.76 [0.64; 0.91]
Random effects model (HK)
0.76 [0.50; 1.16]
Heterogeneity: I^2 = 0%, τ^2 = 0, p = 0.7119
Strategy_group = sacTMT
OptiTROP-Lung04 — sacTMT vs Chemo
0.60 [0.44; 0.82]
Common effect model
0.82 [0.75; 0.90]
Random effects model (HK)
0.82 [0.74; 0.91]
Heterogeneity: I^2 = 3.7%, τ^2 < 0.0001, p = 0.4056
0.1
0.25
0.5
1
2
Hazard Ratio (log scale)

Figure S3. Convergence diagnostics of the Bayesian network meta-analysis for progression-free survival.

Chemo= platinum-based doublet chemotherapy. ICI=immune checkpoint inhibitor. Chemo_ICI=chemotherapy plus ICI. Chemo_Antiangio=chemotherapy plus antiangiogenesis. Chemo_ICI_antiangio=chemotherapy plus antiangiogenesis plus ICI. Chemo_Ivo=chemotherapy plus Ivonescimab. sacTMT= Sacituzumab Tirumotecan. Chemo_Ami=chemotherapy plus Amivantamab. Chemo_Ami_Laz=chemotherapy plus Amivantamab plus lazertinib.

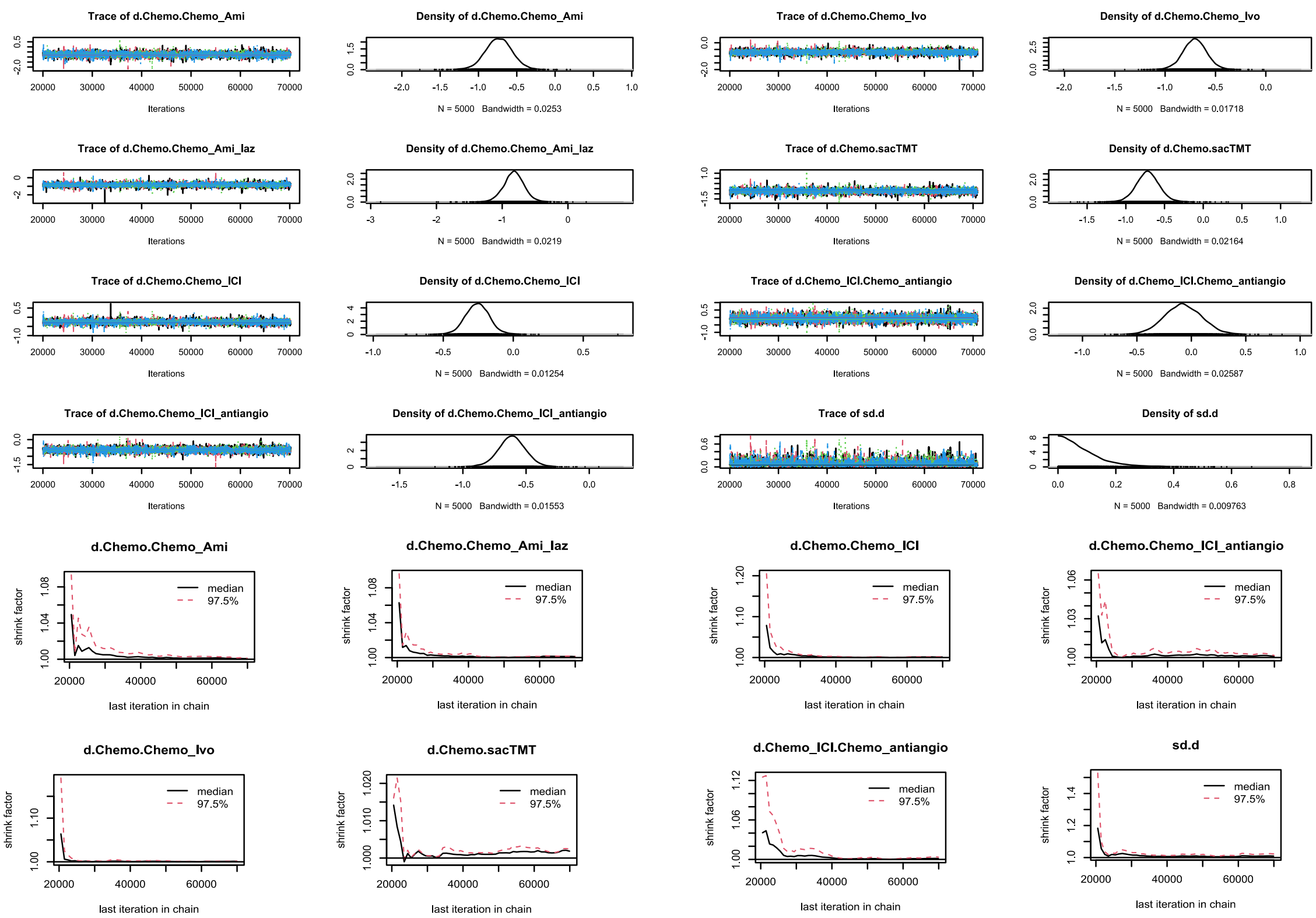

Figure S4. Comparison-adjusted funnel plot for progression-free survival.

Chemo= platinum-based doublet chemotherapy. ICI=immune checkpoint inhibitor. Chemo_ICI=chemotherapy plus ICI. Chemo_Antiangio=chemotherapy plus antiangiogenesis. Chemo_ICI_antiangio=chemotherapy plus antiangiogenesis plus ICI. Chemo_Ivo=chemotherapy plus Ivonescimab. sacTMT= Sacituzumab Tirumotecan. Chemo_Ami=chemotherapy plus Amivantamab. Chemo_Ami_Laz=chemotherapy plus Amivantamab plus lazertinib.

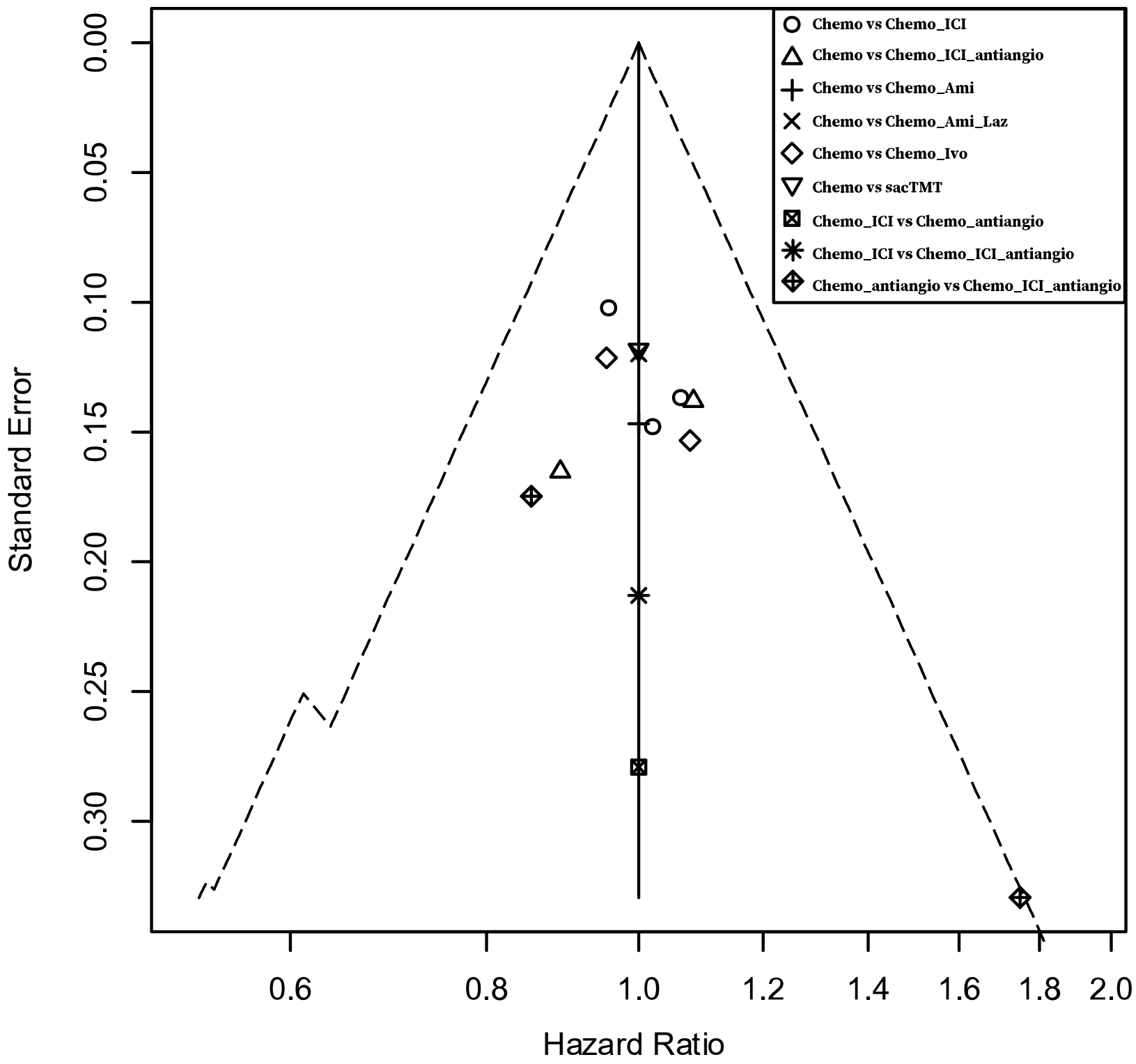

Figure S5. League Table for the network meta-analysis. A) Progression-free survival and Overall survival. B) Objective response rate and Disease control rate. C) Grade≥3 treatment-grade adverse events.

Data are pooled HR (95% credible interval) for A and OR (95% credible interval) for B and C. Bold data indicate a signiffcant difference.

Chemo= platinum-based doublet chemotherapy. ICI=immune checkpoint inhibitor. Chemo_ICI=chemotherapy plus ICI. Chemo_Antiangio=chemotherapy plus antiangiogenesis. Chemo_ICI_antiangio=chemotherapy plus antiangiogenesis plus ICI. Chemo_Ivo=chemotherapy plus Ivonescimab. sacTMT= Sacituzumab Tirumotecan. Chemo_Ami=chemotherapy plus Amivantamab. Chemo_Ami_Laz=chemotherapy plus Amivantamab plus lazertinib.

A

Overall survival (upper right); Progression-free survival (lower left)

Chemo	0.91 (0.76, 1.13)	1.01 (0.67, 1.49)	0.87 (0.67, 1.13)	0.73 (0.49, 1.08)	0.96 (0.62, 1.47)	**0.76 (0.60, 0.98)**	**0.60 (0.41, 0.90)**
**1.29 (1.08, 1.55)**	Chemo_ICI	1.10 (0.73, 1.62)	0.96 (0.70, 1.25)	0.80 (0.51, 1.22)	1.05 (0.65, 1.66)	0.84 (0.60, 1.12)	0.66 (0.42, 1.01)
1.39 (0.97, 1.97)	1.08 (0.75, 1.52)	Chemo_antiangio	0.87 (0.60, 1.24)	0.73 (0.42, 1.27)	0.96 (0.53, 1.73)	0.76 (0.48, 1.22)	0.60 (0.34, 1.06)
**1.85 (1.48, 2.32)**	**1.43 (1.11, 1.84)**	1.33 (0.99, 1.82)	Chemo_ICI_antiangio	0.83 (0.53, 1.35)	1.10 (0.67, 1.83)	0.88 (0.62, 1.26)	0.69 (0.43, 1.12)
**2.09 (1.45, 3.02)**	**1.61 (1.07, 2.44)**	1.49 (0.91, 2.53)	1.13 (0.74, 1.72)	Chemo_Ami	1.32 (0.74, 2.33)	1.05 (0.66, 1.65)	0.83 (0.47, 1.46)
**2.27 (1.63, 3.11)**	**1.76 (1.21, 2.54)**	**1.63 (1.02, 2.66)**	1.23 (0.82, 1.82)	1.09 (0.67, 1.77)	Chemo_Ami_laz	0.80 (0.49, 1.31)	0.63 (0.35, 1.12)
**2.02 (1.58, 2.61)**	**1.56 (1.16, 2.15)**	1.45 (0.95, 2.27)	1.09 (0.78, 1.54)	0.97 (0.63, 1.52)	0.89 (0.60, 1.35)	Chemo_Ivo	0.79 (0.49, 1.26)
**2.04 (1.48, 2.83)**	**1.58 (1.10, 2.29)**	1.47 (0.91, 2.40)	1.11 (0.74, 1.64)	0.98 (0.60, 1.57)	0.90 (0.57, 1.43)	1.01 (0.67, 1.52)	sacTMT

B

Disease control rate (upper right); Objective response rate (lower left)

Chemo	1.49 (0.99, 2.39)	2.15 (0.66, 7.94)	**2.24 (1.26, 4.29)**	**3.15 (1.42, 7.28)**	**2.86 (1.36, 6.05)**	**2.13 (1.20, 3.89)**	1.67 (0.76, 3.73)
0.80 (0.53–1.20)	Chemo_ICI	1.44 (0.45, 5.08)	1.50 (0.82, 2.79)	2.11 (0.83, 5.27)	1.93 (0.78, 4.34)	1.43 (0.68, 2.90)	1.12 (0.43, 2.77)
0.48 (0.23–1.06)	0.60 (0.30, 1.28)	Chemo_antiangio	1.05 (0.29, 3.47)	1.46 (0.32, 6.18)	1.33 (0.29, 5.36)	0.99 (0.24, 3.80)	0.77 (0.17, 3.28)
**0.41 (0.25–0.66)**	**0.52 (0.33, 0.81)**	0.87 (0.43, 1.60)	Chemo_ICI_antiangio	1.41 (0.49, 3.80)	1.29 (0.47, 3.20)	0.95 (0.40, 2.14)	0.75 (0.26, 1.99)
**0.32 (0.15–0.71)**	**0.40 (0.16, 0.99)**	0.67 (0.21, 1.94)	0.78 (0.31, 2.00)	Chemo_Ami	0.91 (0.39, 2.09)	0.68 (0.25, 1.81)	0.53 (0.17, 1.65)
**0.34 (0.16–0.72)**	0.43 (0.19, 1.01)	0.72 (0.24, 2.00)	0.83 (0.35, 2.05)	1.06 (0.50, 2.26)	Chemo_Ami_laz	0.74 (0.30, 1.91)	0.58 (0.20, 1.72)
0.59 (0.35–1.01)	0.74 (0.38, 1.48)	1.24 (0.47, 3.04)	1.43 (0.70, 3.00)	1.84 (0.71, 4.83)	1.74 (0.69, 4.34)	Chemo_Ivo	0.79 (0.29, 2.10)
0.49 (0.23–1.02)	0.61 (0.26, 1.45)	1.03 (0.33, 2.77)	1.19 (0.49, 2.89)	1.52 (0.51, 4.39)	1.43 (0.49, 4.01)	0.82 (0.32, 2.10)	sacTMT

C

Grade≥3 Treatment-Related Adverse Events

Chemo							
0.79 (0.39–1.42)	Chemo_ICI						
0.77 (0.20–2.87)	0.98 (0.29, 3.48)	Chemo_antiangio					
0.51 (0.23–1.04)	0.65 (0.30, 1.39)	0.66 (0.18, 2.39)	Chemo_ICI_antiangio				
**0.27 (0.09–0.87)**	0.34 (0.10, 1.34)	0.35 (0.06, 2.07)	0.53 (0.14, 2.19)	Chemo_Ami			
**0.08 (0.03–0.27)**	**0.11 (0.03, 0.41)**	**0.11 (0.02, 0.62)**	**0.16 (0.04, 0.66)**	0.31 (0.10, 1.02)	Chemo_Ami_laz		
0.67 (0.30–1.48)	0.85 (0.31, 2.51)	0.86 (0.18, 4.07)	1.30 (0.44, 4.06)	2.49 (0.60, 9.89)	**8.05 (1.94, 32.36)**	Chemo_Ivo	
0.84 (0.27–2.63)	1.06 (0.30, 4.18)	1.08 (0.20, 6.59)	1.64 (0.46, 6.75)	3.15 (0.60, 16.04)	**10.19 (1.95, 52.39)**	1.27 (0.32, 5.12)	sacTMT

Figure S6. Efficacy and Safety Rankings Based on Cumulative Ranking Probabilities. A) Progression-free survival. B) Overall survival. C) Objective response rate. D) Grade ⩾ 3 treatment-grade adverse events.

Stacked bars show the cumulative probabilities of each treatment achieving different ranks. Higher probabilities of better ranks indicate greater efficacy. For grade ≥3 adverse events, rank 1 represents the safest treatment, and higher probabilities of better ranks indicate lower toxicity.

Chemo= platinum-based doublet chemotherapy. ICI=immune checkpoint inhibitor. Chemo_ICI=chemotherapy plus ICI. Chemo_Antiangio=chemotherapy plus antiangiogenesis. Chemo_ICI_antiangio=chemotherapy plus antiangiogenesis plus ICI. Chemo_Ivo=chemotherapy plus Ivonescimab. sacTMT= Sacituzumab Tirumotecan. Chemo_Ami=chemotherapy plus Amivantamab. Chemo_Ami_Laz=chemotherapy plus Amivantamab plus lazertinib.

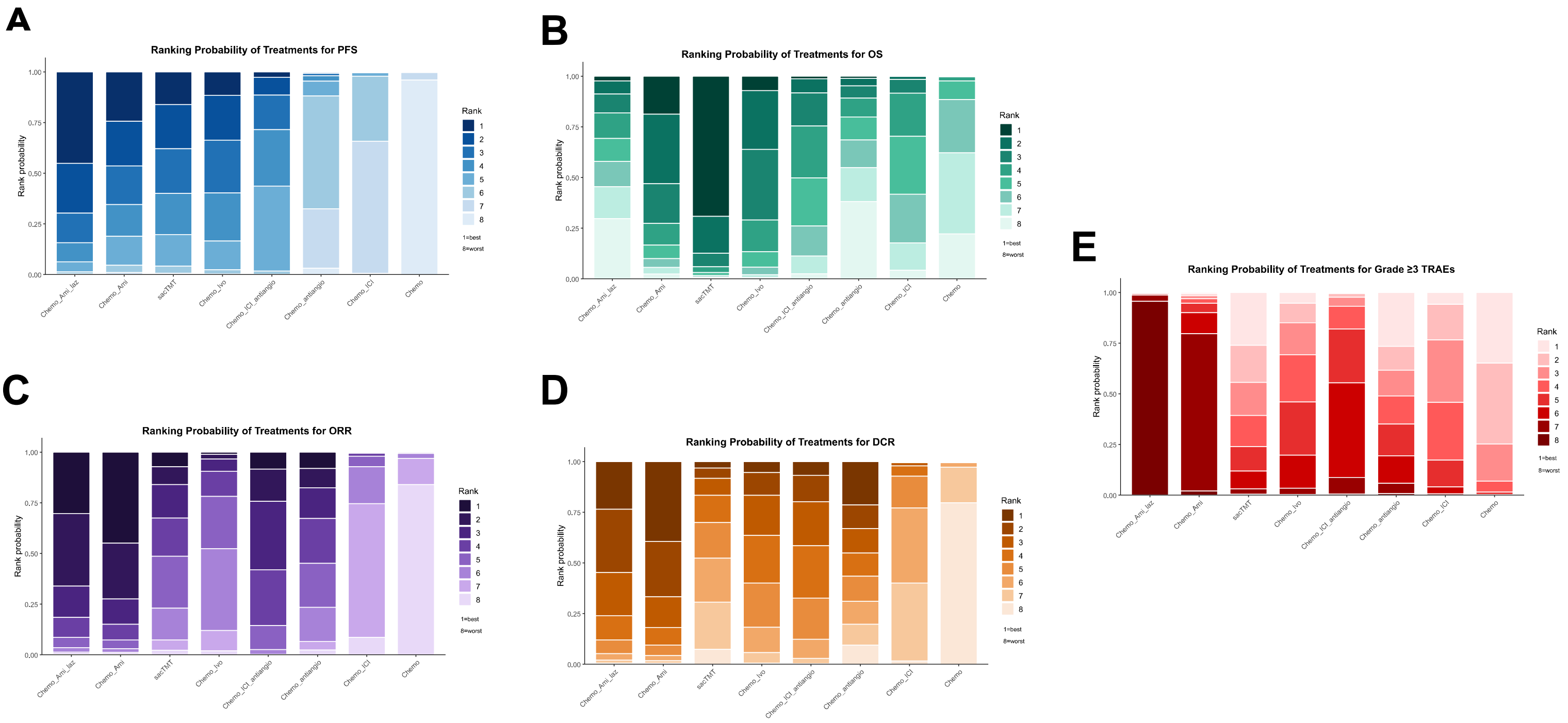

Figure S7. Efficacy and safety rankings Based on SUCRA. A) Progression-free survival. B) Overall survival. C) Objective response rate. D) Disease control rate. E) Grade⩾3 treatment-grade adverse events.

Chemo= platinum-based doublet chemotherapy. ICI=immune checkpoint inhibitor. Chemo_ICI=chemotherapy plus ICI. Chemo_Antiangio=chemotherapy plus antiangiogenesis. Chemo_ICI_antiangio=chemotherapy plus antiangiogenesis plus ICI. Chemo_Ivo=chemotherapy plus Ivonescimab. sacTMT= Sacituzumab Tirumotecan. Chemo_Ami=chemotherapy plus Amivantamab. Chemo_Ami_Laz=chemotherapy plus Amivantamab plus lazertinib.

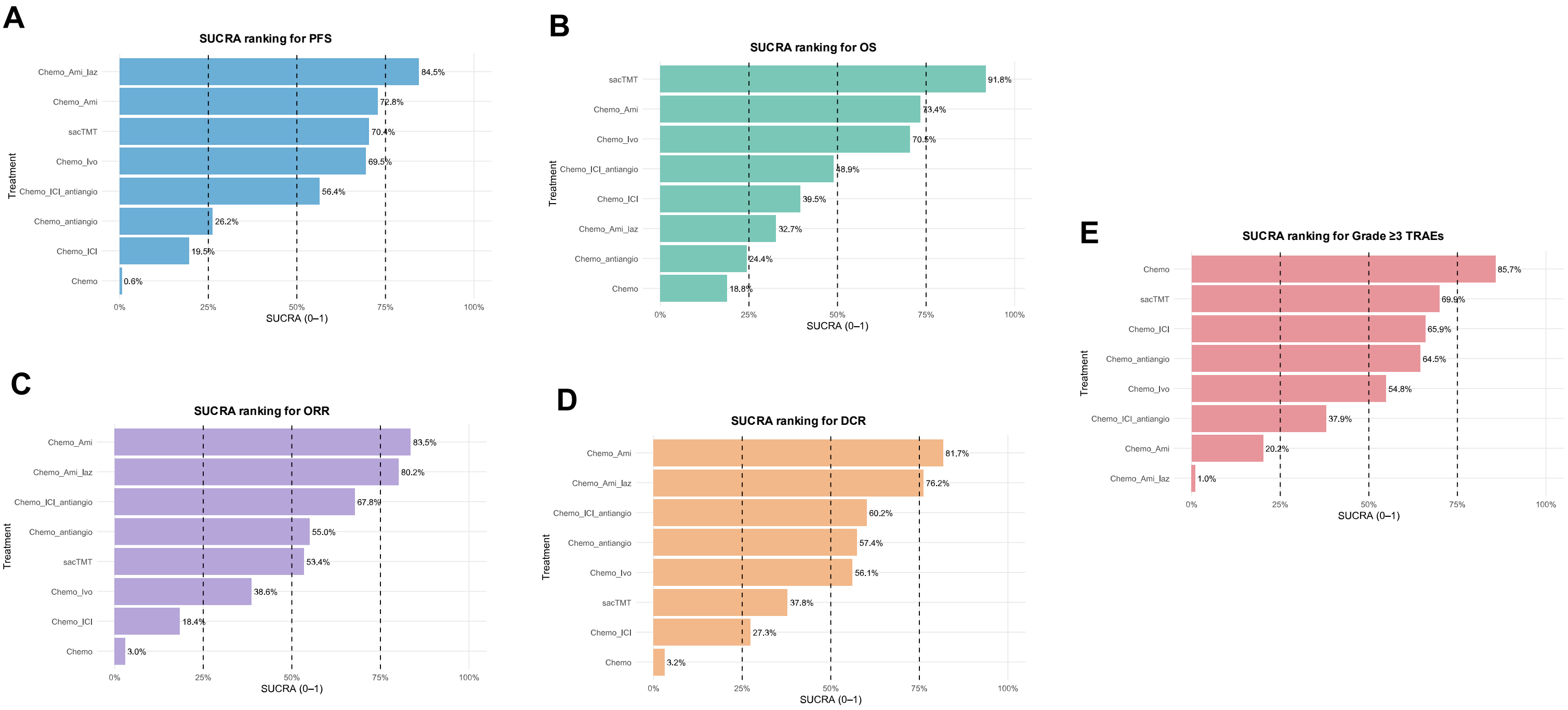

Figure S8. Sensitivity analysis for grade ≥3 TRAEs excluding the TEAE-reporting trial (HARMONi-A). A) Forest plot. B) SUCRA.

*For visualization purposes, the upper limit of the 95% credible interval was capped at 13.00. Chemo= platinum-based doublet chemotherapy. ICI=immune checkpoint inhibitor. Chemo_ICI=chemotherapy plus ICI. Chemo_Antiangio=chemotherapy plus antiangiogenesis. Chemo_ICI_antiangio=chemotherapy plus antiangiogenesis plus ICI. Chemo_Ivo=chemotherapy plus Ivonescimab. sacTMT= Sacituzumab Tirumotecan. Chemo_Ami=chemotherapy plus Amivantamab. Chemo_Ami_Laz=chemotherapy plus Amivantamab plus lazertinib.

A

B

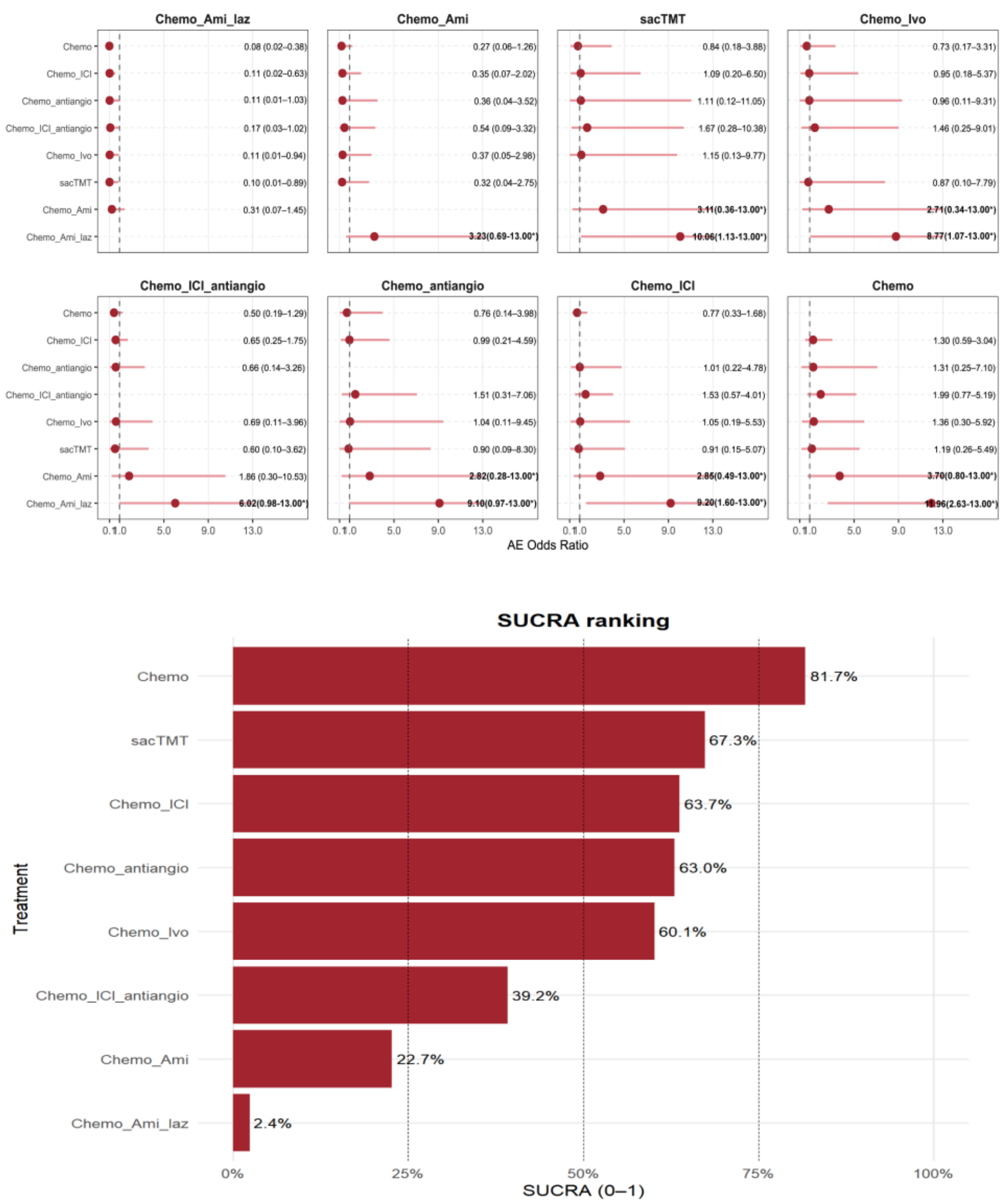

Figure S9. League tables of key safety endpoints. A) Serious adverse event. B) Treatment-related deaths. C) Treatment discontinuation. D) Anemia. E) Decreased WBC count. F) Decreased platelet count.

NE: Not Estimable. Due to zero events reported in the sacTMT group within the included trials, the corresponding statistical estimates are not estimable.

Across the 11 RCTs, 9 informed the overall safety analysis. For the specific sub-endpoints A–F, the number of included RCTs was 7, 7, 8, 7, 6, and 6, respectively.

Chemo= platinum-based doublet chemotherapy. ICI=immune checkpoint inhibitor. Chemo_ICI=chemotherapy plus ICI. Chemo_Antiangio=chemotherapy plus antiangiogenesis. Chemo_ICI_antiangio=chemotherapy plus antiangiogenesis plus ICI. Chemo_Ivo=chemotherapy plus Ivonescimab. sacTMT= Sacituzumab Tirumotecan. Chemo_Ami=chemotherapy plus Amivantamab. Chemo_Ami_Laz=chemotherapy plus Amivantamab plus lazertinib.

A

Serious adverse event (SAE)

Chemo							
0.48 (0.06, 3.09)	Chemo_ICI						
0.92 (0.03, 20.36)	1.93 (0.10, 34.86)	Chemo_antiangio					
0.25 (0.03, 1.66)	0.52 (0.07, 3.69)	0.27 (0.01, 4.82)	Chemo_ICI_antiangio				
0.31 (0.01, 6.90)	0.65 (0.02, 29.83)	0.34 (0.00, 37.58)	1.25 (0.04, 61.36)	Chemo_Ami			
0.13 (0.01, 2.77)	0.27 (0.01, 12.36)	0.14 (0.00, 15.80)	0.51 (0.02, 25.48)	0.41 (0.02, 9.34)	Chemo_Ami_laz		
0.49 (0.02, 10.57)	1.01 (0.03, 44.18)	0.52 (0.01, 64.04)	1.94 (0.06, 96.33)	1.55 (0.02, 127.66)	3.68 (0.05, 276.14)	Chemo_Ivo	
2.16 (0.10, 47.38)	4.51 (0.12, 195.63)	2.35 (0.03, 250.94)	8.59 (0.25, 457.71)	6.94 (0.09, 574.78)	16.61 (0.21, 1288.69)	4.51 (0.05, 307.24)	sacTMT

B

Treatment-related deaths

Chemo						
0.90 (0.10, 6.95)	Chemo_ICI					
**0.10 (0.01, 0.69)**	**0.11 (0.01, 0.92)**	Chemo_ICI_antiangio				
0.24 (0.01, 4.46)	0.26 (0.00, 9.67)	2.50 (0.04, 123.00)	Chemo_Ami			
0.21 (0.01, 2.61)	0.23 (0.00, 6.07)	2.14 (0.04, 85.20)	0.89 (0.07, 8.40)	Chemo_Ami_laz		
0.84 (0.24, 3.36)	0.93 (0.09, 12.11)	8.78 (0.92, 164.17)	3.60 (0.15, 168.19)	4.03 (0.25, 183.82)	Chemo_Ivo	
NE	NE	NE	NE	NE	NE	sacTMT

C

Treatment discontinuation

Chemo					
0.60 (0.32, 1.05)	Chemo_ICI				
0.71 (0.18, 2.70)	1.19 (0.35, 4.29)	Chemo_antiangio			
**0.24 (0.09, 0.50)**	**0.40 (0.18, 0.80)**	0.33 (0.09, 1.10)	Chemo_ICI_antiangio		
0.63 (0.27, 1.36)	1.06 (0.38, 2.83)	0.88 (0.18, 4.21)	2.66 (0.88, 8.86)	Chemo_Ivo	
NE	NE	NE	NE	NE	sacTMT

D **Anemia**

Chemo						
0.91 (0.59, 1.39)	Chemo_ICI					
1.12 (0.65, 2.04)	1.23 (0.69, 2.37)	Chemo_ICI_antiangio				
1.09 (0.51, 2.36)	1.20 (0.51, 2.91)	0.98 (0.36, 2.44)	Chemo_Ami			
0.55 (0.27, 1.13)	0.60 (0.26, 1.40)	0.49 (0.19, 1.17)	0.50 (0.23, 1.06)	Chemo_Ami_laz		
0.81 (0.20, 3.20)	0.89 (0.21, 3.79)	0.72 (0.16, 3.15)	0.73 (0.16, 3.54)	1.47 (0.32, 6.96)	Chemo_Ivo	
0.58 (0.26, 1.32)	0.64 (0.26, 1.60)	0.52 (0.19, 1.34)	0.53 (0.18, 1.60)	1.06 (0.37, 3.16)	0.72 (0.15, 3.53)	sacTMT

E **Decreased WBC count**

Chemo						
0.99 (0.58, 1.63)	Chemo_ICI					
1.14 (0.50, 2.52)	1.15 (0.52, 2.63)	Chemo_ICI_antiangio				
0.97 (0.39, 2.32)	0.98 (0.34, 2.76)	0.85 (0.25, 2.84)	Chemo_Ami			
0.62 (0.26, 1.43)	0.63 (0.23, 1.66)	0.55 (0.17, 1.70)	0.64 (0.26, 1.54)	Chemo_Ami_laz		
1.81 (0.36, 10.68)	1.84 (0.34, 11.55)	1.60 (0.26, 11.21)	1.92 (0.29, 13.55)	2.97 (0.47, 20.25)	Chemo_Ivo	
0.45 (0.18, 1.12)	0.46 (0.16, 1.33)	0.40 (0.12, 1.36)	0.47 (0.13, 1.72)	0.73 (0.21, 2.61)	0.25 (0.03, 1.60)	sacTMT

F **Decreased platelet count**

Chemo						
1.01 (0.46, 2.34)	Chemo_ICI					
0.54 (0.16, 2.02)	0.54 (0.15, 1.97)	Chemo_ICI_antiangio				
0.48 (0.12, 1.91)	0.48 (0.10, 2.22)	0.89 (0.14, 5.56)	Chemo_Ami			
0.26 (0.07, 1.02)	0.26 (0.05, 1.17)	0.48 (0.07, 2.95)	0.55 (0.14, 2.14)	Chemo_Ami_laz		
0.42 (0.08, 2.04)	0.42 (0.07, 2.44)	0.78 (0.10, 5.90)	0.88 (0.11, 7.09)	1.62 (0.19, 12.29)	Chemo_Ivo	
2.03 (0.50, 7.77)	2.01 (0.38, 9.27)	3.73 (0.55, 23.36)	4.21 (0.62, 28.64)	**7.65 (1.13, 53.95)**	4.78 (0.59, 40.25)	sacTMT

Figure S10. GRADE certainty of evidence. A) Progression-free survival. B) Overall survival. C) Objective response rate. D) Disease control rate. E) Grade≥3 treatment-grade adverse events.

Chemo= platinum-based doublet chemotherapy. ICI=immune checkpoint inhibitor. Chemo_ICI=chemotherapy plus ICI. Chemo_Antiangio=chemotherapy plus antiangiogenesis. Chemo_ICI_antiangio=chemotherapy plus antiangiogenesis plus ICI. Chemo_Ivo=chemotherapy plus Ivonescimab. sacTMT= Sacituzumab Tirumotecan. Chemo_Ami=chemotherapy plus Amivantamab. Chemo_Ami_Laz=chemotherapy plus Amivantamab plus lazertinib.

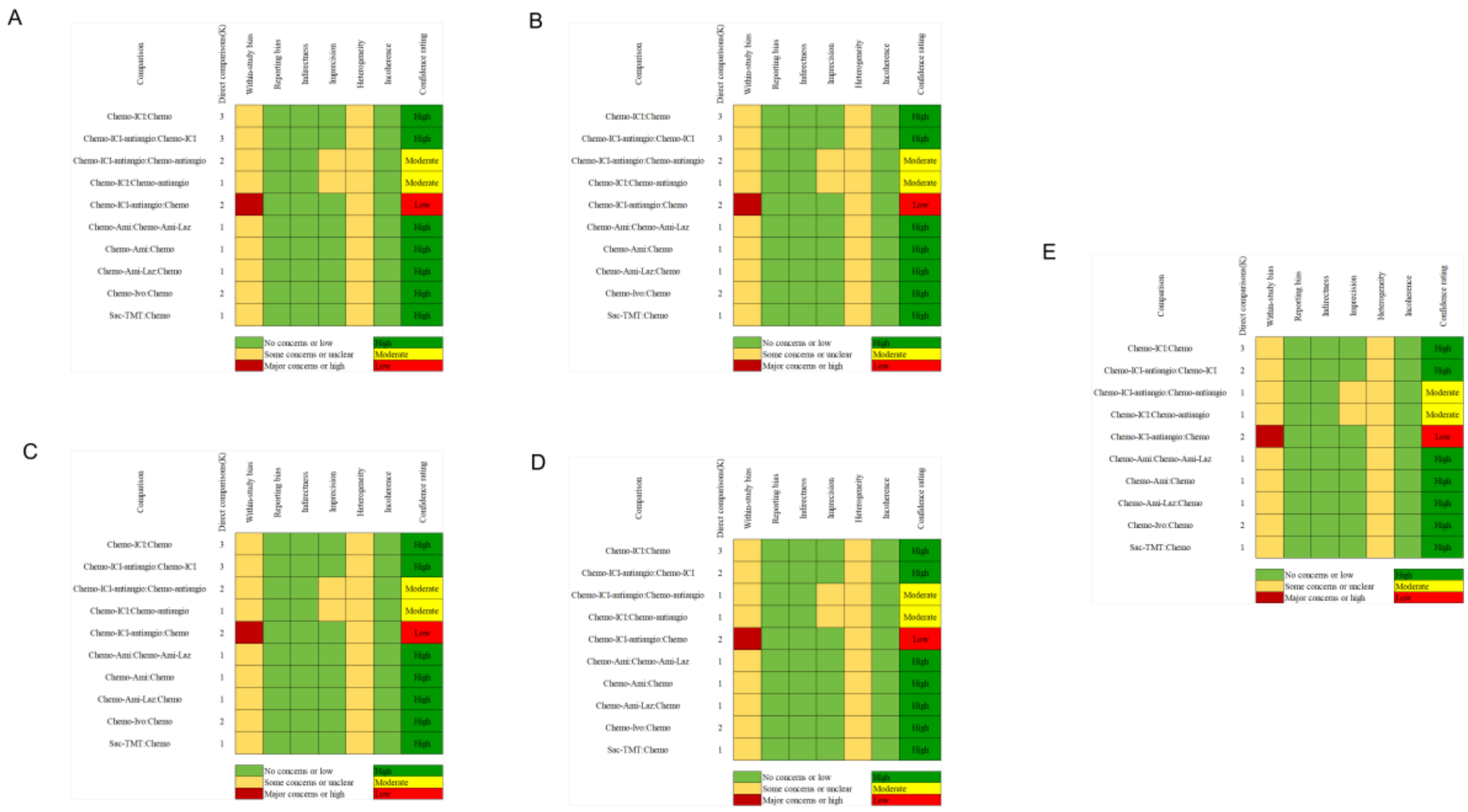

References

1. Mok, T., et al., *Nivolumab Plus Chemotherapy in Epidermal Growth Factor Receptor-Mutated Metastatic Non-Small-Cell Lung Cancer After Disease Progression on Epidermal Growth Factor Receptor Tyrosine Kinase Inhibitors: Final Results of CheckMate 722.* J Clin Oncol, 2024. **42**(11): p. 1252-1264.
2. Yang, J.C., et al., *Phase III KEYNOTE-789 Study of Pemetrexed and Platinum With or Without Pembrolizumab for Tyrosine Kinase Inhibitor–Resistant, EGFR-Mutant, Metastatic Nonsquamous Non-Small Cell Lung Cancer.* J Clin Oncol, 2024. **42**(34): p. 4029-4039.
3. Reck, M., et al., *Atezolizumab plus bevacizumab and chemotherapy in non-small-cell lung cancer (IMpower150): key subgroup analyses of patients with EGFR mutations or baseline liver metastases in a randomised, open-label phase 3 trial.* Lancet Respir Med, 2019. **7**(5): p. 387-401.
4. Nogami, N., et al., *IMpower150 Final Exploratory Analyses for Atezolizumab Plus Bevacizumab and Chemotherapy in Key NSCLC Patient Subgroups With EGFR Mutations or Metastases in the Liver or Brain.* J Thorac Oncol, 2022. **17**(2): p. 309-323.
5. Lu, S., et al., *Sintilimab plus bevacizumab biosimilar IBI305 and chemotherapy for patients with EGFR-mutated non-squamous non-small-cell lung cancer who progressed on EGFR tyrosine-kinase inhibitor therapy (ORIENT-31): first interim results from a randomised, double-blind, multicentre, phase 3 trial.* Lancet Oncol, 2022. **23**(9): p. 1167-1179.
6. Lu, S., et al., *Sintilimab plus chemotherapy for patients with EGFR-mutated non-squamous non-small-cell lung cancer with disease progression after EGFR tyrosine-kinase inhibitor therapy (ORIENT-31): second interim analysis from a double-blind, randomised, placebo-controlled, phase 3 trial.* Lancet Respir Med, 2023. **11**(7): p. 624-636.
7. Park, S., et al., *Phase III, Randomized Study of Atezolizumab Plus Bevacizumab and Chemotherapy in Patients With EGFR- or ALK-Mutated Non-Small-Cell Lung Cancer (ATTLAS, KCSG-LU19-04).* J Clin Oncol, 2024. **42**(11): p. 1241-1251.
8. Zhou, C., et al., *Atezolizumab plus bevacizumab and chemotherapy in metastatic nonsquamous NSCLC: the randomized double-blind phase 3 IMpower151 trial.* Nat Med, 2025. **31**(7): p. 2375-2384.
9. Passaro, A., et al., *Amivantamab plus chemotherapy with and without lazertinib in EGFR-mutant advanced NSCLC after disease progression on osimertinib: primary results from the phase III MARIPOSA-2 study.* Ann Oncol, 2024. **35**(1): p. 77-90.
10. Popat, S., et al., *LBA54 Amivantamab plus chemotherapy vs chemotherapy in EGFR-mutated, advanced non-small cell lung cancer after disease progression on osimertinib: Second interim overall survival from MARIPOSA-2.* Annals of Oncology, 2024. **35**: p. S1244-S1245.
11. Long, R., W. Kuang, and Q. Zhou, *Ivonescimab plus chemotherapy in advanced or metastatic non-squamous non-small cell lung cancer with EGFR variant in China: a cost-effectiveness analysis.* Transl Lung Cancer Res, 2025. **14**(5): p. 1622-1634.
12. Zhang, L., et al., *1348 Final overall survival analysis of HARMONi-A study comparing ivonescimab plus chemotherapy to chemotherapy alone in patients with EGFR+ NSCLC progressed on EGFR-TKI treatment.* Journal for ImmunoTherapy of Cancer, 2025. **13**(Suppl 3).
13. Shiraishi, Y., et al., *Atezolizumab and Platinum Plus Pemetrexed With or Without Bevacizumab for Metastatic Nonsquamous Non-Small Cell Lung Cancer: A Phase 3 Randomized Clinical Trial.* JAMA Oncol, 2024. **10**(3): p. 315-324.
14. Fang, W., et al., *Sacituzumab Tirumotecan in EGFR-TKI-Resistant, EGFR-Mutated Advanced NSCLC.* N Engl J Med, 2026. **394**(1): p. 13-26.
15. Goldman, J.W., et al., *PL02.12 Ivonescimab vs Placebo Plus Chemo, Phase 3 in Patients with EGFR+ NSCLC Progressed with 3rd gen EGFR-TKI Treatment: HARMONi.* Journal of Thoracic Oncology, 2025. **20**(10): p. S2-S3.
